# Body mass index had different effects on premenopausal and postmenopausal breast cancer risks: a dose-response meta-analysis with 3,318,796 subjects from 31 cohort studies

**DOI:** 10.1186/s12889-017-4953-9

**Published:** 2017-12-08

**Authors:** Yanzi Chen, Li Liu, Quan Zhou, Mustapha Umar Imam, Jialin Cai, Yaxuan Wang, Minjie Qi, Panpan Sun, Zhiguang Ping, Xiaoli Fu

**Affiliations:** 1 College of Public Health, Zhengzhou University, Zhengzhou, Henan China; 2 School of Basic Medical Sciences, Zhengzhou University, Zhengzhou, Henan China; 3 Department of Science and Education, The First People’s Hospital of Changde City, Changde, Hunan China

**Keywords:** Body mass index (BMI), Breast cancer, Dose-response relationship, Meta-analysis, Cohort study

## Abstract

**Background:**

There is sufficient evidence supporting a relationship between increased body mass index (BMI) and an increased risk for breast cancer among postmenopausal women. However, most studies have found a decreased risk for premenopausal breast cancer. This study was conducted to find out the different effects of BMI on the risk of breast cancer among premenopausal and postmenopausal women, and explore the potential factors that influence the associations.

**Methods:**

A dose-response meta-analysis with 3,318,796 participants from 31 articles was conducted. Cohort studies that included BMI and corresponding breast cancer risk were selected through various databases including PubMed, Medline, Web of Science, the China National Knowledge Infrastructure (CNKI) and Chinese Scientific Journals (VIP). Random effects models were used for analyzing the data.

**Results:**

The summary relative risks (RRs) were 1.33 (95%CI: 1.20–1.48) and 0.94(95%CI: 0.80–1.11) among postmenopausal and premenopausal women, respectively. The dose-response meta-analysis indicated a positive non-linear association between BMI and breast cancer risk among postmenopausal women, and compared to the mean level of the normal BMI category (21.5 kg/m^2^) the RR in total postmenopausal women were1.03 (95% CI: 1.02–1.05) per 1 kg/m^2^ increment. However, no statistically significant association among total premenopausal women was detected. In subgroup analysis among European premenopausal women, the summary RR was 0.79(95%CI: 0.70–0.88). The non-linear relationship showed a negative non-linear association between BMI and breast cancer risk among European premenopausal women. When compared to the mean level of the normal BMI category, the RRs were 0.98 (95%CI: 0.96–1.00) per 1 kg/m^2^ increment, respectively.

**Conclusions:**

In line with previous studies BMI had different effects on pre-menopausal and postmenopausal breast cancer risk. However, contrary to previous studies, a high BMI was not associated with decreased risk in total pre-menopausal women. More research is needed to better understand these differences.

**Electronic supplementary material:**

The online version of this article (10.1186/s12889-017-4953-9) contains supplementary material, which is available to authorized users.

## Background

Breast cancer is the most frequently diagnosed cancer and the leading cause of cancer death among females worldwide. It accounts for 25% of all cancer cases and 15% of all cancer deaths among females [1]. Breast cancer is a known health consequence of overweight and obesity [2].

There is sufficient evidence supporting a relationship between increased body mass index (BMI) and an increased risk for breast cancer among postmenopausal women [3–5]_._ However, studies among premenopausal women are inconsistent and unclear. Some studies have shown a decreased risk for premenopausal breast cancer [5–7], while others have suggested no association [3, 8, 9]. The American Institute for Cancer Research (AICR) & World Cancer Research Fund (WCRF) continuous updated report on nutrition and cancer [10] currently defines higher BMI as a convincing risk factor for postmenopausal breast cancer while for premenopausal women higher BMI is defined as probably decreasing breast cancer risk. The present meta-analysis which may included more cohort articles and subgroup analyses was conducted to find out the different effects of BMI on the risks of breast cancer among premenopausal and postmenopausal women, and explore other potential factors that influence the associations deeply.

## Methods and materials

### Search strategy

We searched PubMed, Medline, Web of Science, and Chinese academic databases including the China National Knowledge Infrastructure (CNKI) and Chinese Scientific Journals (VIP) databases for publications on the associations between BMI and breast cancer in humans, through December 31, 2015. The keywords were (obesity OR obese OR adiposity OR fat OR fatness OR “body mass index” OR BMI OR “body size” OR weight OR overweight) AND (“breast cancer” OR “breast carcinoma” OR “breast neoplasm”). References from reviews and meta-analyses were also searched for additional publications.

### Study selection and data extraction

Studies were included according to the following criteria:

1. Original article publication.
2. Study design was a prospective cohort study.
3. Study had BMI categories of no fewer than three, with a relative risk (RR) or hazard ratio (HR), and corresponding 95% confidence interval (CI) for the breast cancer presented for each BMI category.
4. The studies provided the cases and person-years number for each BMI category.

From the selected studies the information extracted was: first author’s last name; publication year; country of origin; duration of follow-up; intervals of each BMI category; number of cases and cohort size of each category; relative risks and the 95% CI for each BMI category; and variables adjusted for in the multivariable analysis. The results that were adjusted by the most possible confounding factors were extracted. If data were reported by age category, hormone replacement therapy (HRT) use or other factors in one publication, they were considered as different studies. Two investigators (Cai and Wang) independently retrieved the data. The disagreement was resolved by mutual discussion.

**Fig. 1:**
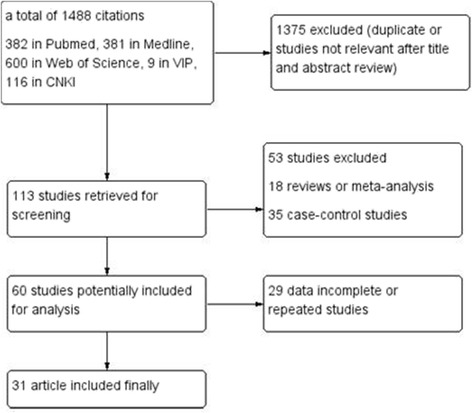
Flow chart showing literature retrieval and selection for this meta-analysis (CNKI (China National Knowledge Infrastructure) and VIP (Chinese Scientific Journals) are Chinese academic databases)

Figure 1 shows the flow chart for the literature retrieval and selection. During the first round of the search, a total of 1488 published articles from PubMed, Web of Science, Medline and Chinese academic (CNKI and VIP) databases prior to December 31, 2015 were identified. All the identified articles were screened, after which only 31 papers were selected. Other studies were excluded due to the following reasons:

1. 1375 studies were duplicated or irrelevant to our study according to titles or abstracts.
2. 18 studies were reviews or meta-analyses.
3. 35 studies were case-control studies.
4. 29 studies were excluded because the data needed were not available.

### Quality assessment of studies

Using the Newcastle-Ottawa Scale (NOS), the quality of the included studies was assessed independently by two reviewers (Cai and Wang). This scale ranges from 0 to 9 stars and awards four stars for selection of study participants, two stars for comparability of studies, and three stars for the adequate ascertainment of outcomes. Scores of 0–3, 4–6, and 7–9 are viewed as low, moderate, and high quality, respectively.

### Statistical analysis

Because the incidence of breast cancer is low, HR is mathematically similar to RR. The results were reported as RR. The associations between BMI and the risk of breast cancer were quantified by comparing the highest versus the lowest categories. For the dose-response meta-analysis, the dosage value corresponding to each BMI was the median of the upper and lower boundaries. For the studies that did not report a median, the midpoint of closed categories was used. In the case of open ended highest category or lowest category, we assumed that the boundary had the same amplitude as the adjacent category [11, 12].

The least squares estimation was conducted to account for the correlation with the logRR estimates across the BMI [13, 14]. The non-linear dose-response relationship was estimated in two stages [15]: firstly, the distribution of BMI was estimated using the restricted cubic spline model with 3 knots at percentiles 10%, 50% and 90%, and the 2 regression coefficients calculated; secondly, the variance or covariance matrix within each study was combined. A non-linearity test was conducted, by testing the null hypothesis that the coefficient of the second spline is equal to zero.

Heterogeneity among studies was assessed using the *χ*2 test. The quantification of heterogeneity was assessed by the *I* ^2^ statistic. An *I* ^2^ above 50% indicated high heterogeneity. A random effect model was implemented. Publication bias was identified with the Begg’s rank correlation test and Egger’s regression test [16, 17]. The highest versus the lowest BMI meta-analysis and subgroup analysis were performed in Review Manager 5.3 (Cochrane). The sensitivity analyses and trim-and-fill analysis [18] were estimated in Stata 12.0. The non-linear dose-response meta-analysis was conducted in R 3.2.0. Statistical significance was present when *P* < 0.05, except where there was a publication bias or heterogeneity when *P* < 0.10.

## Results

### Study characteristics

Additional file 1 shows the characteristics of all the selected studies. All 31 selected studies were prospective cohort, with a total of 3,318,796 participants included in this meta-analysis. A total of 42,271 cases of breast cancer were documented during the follow-up. Among the 31 articles, six studies provided information on all women included in the study not minding their menopausal status [19–24], 17 studies provided information on premenopausal and postmenopausal women independently [3, 7, 8, 22–35], ten studies provided data on postmenopausal women only [4, 36–44], and one study provided information about premenopausal women only [45].

Among the studies, 12 were conducted in Europe [3, 7, 8, 20, 24, 28, 29, 34, 36, 40, 41, 45], 12 in the USA [4, 19, 22, 26, 30–32, 37, 39, 42–44], six in Asia [23, 25, 27, 33, 35, 38] and one in Australia [21]. In addition, eight studies provided the data on HRT non-users [3, 4, 30, 34, 39, 41, 42, 44], while four studies provided the data on HRT ever-users [3, 4, 34, 41]. Furthermore, six studies provided data on estrogen receptor positive breast cancer [26, 30, 39, 42–44], while five studies provided estrogen receptor negative data [26, 30, 42–44]. Thus, we conducted subgroup analyses to identify possible sources of heterogeneity. The participants’ age at baseline ranged from 30 to 79 years old, and all studies provided adjusted risk estimates; the RRs were adjusted for age, smoking, height, alcohol, physical activity, age at menarche, education, parity, marital status, use of HRT, family history of breast cancer, study area and other factors. NOS scores ranged from six to nine.

### Highest versus lowest BMI meta-analysis

**Fig. 2:**
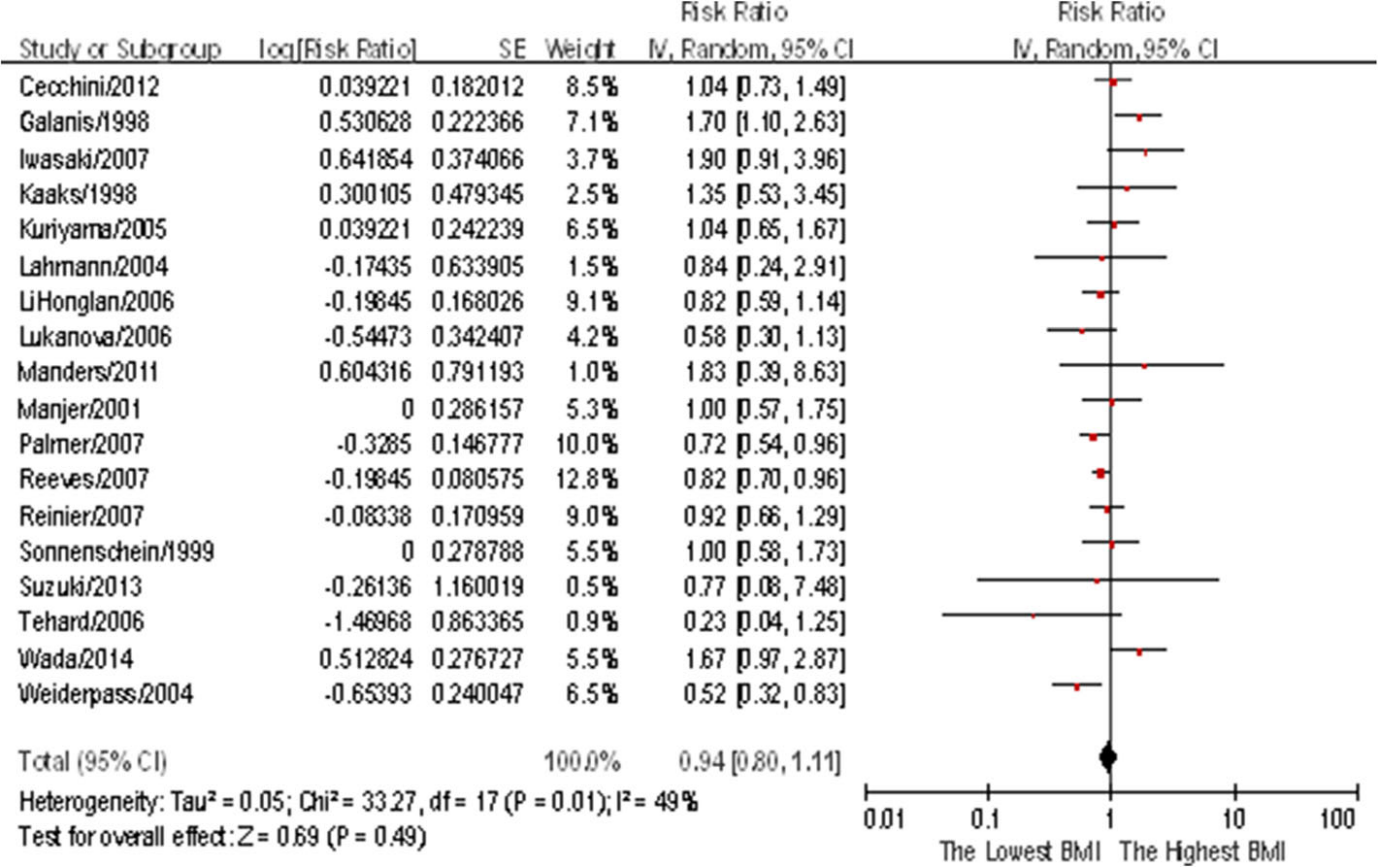
Forest plot of body mass index (BMI) and relative risk of breast cancer in premenopausal women (The highest vs. lowest BMI categories are being compared, the pooled RR was 0.94 (0.80–1.11), which showed no association between BMI and breast cancer risk in premenopausal women)

**Fig. 3:**
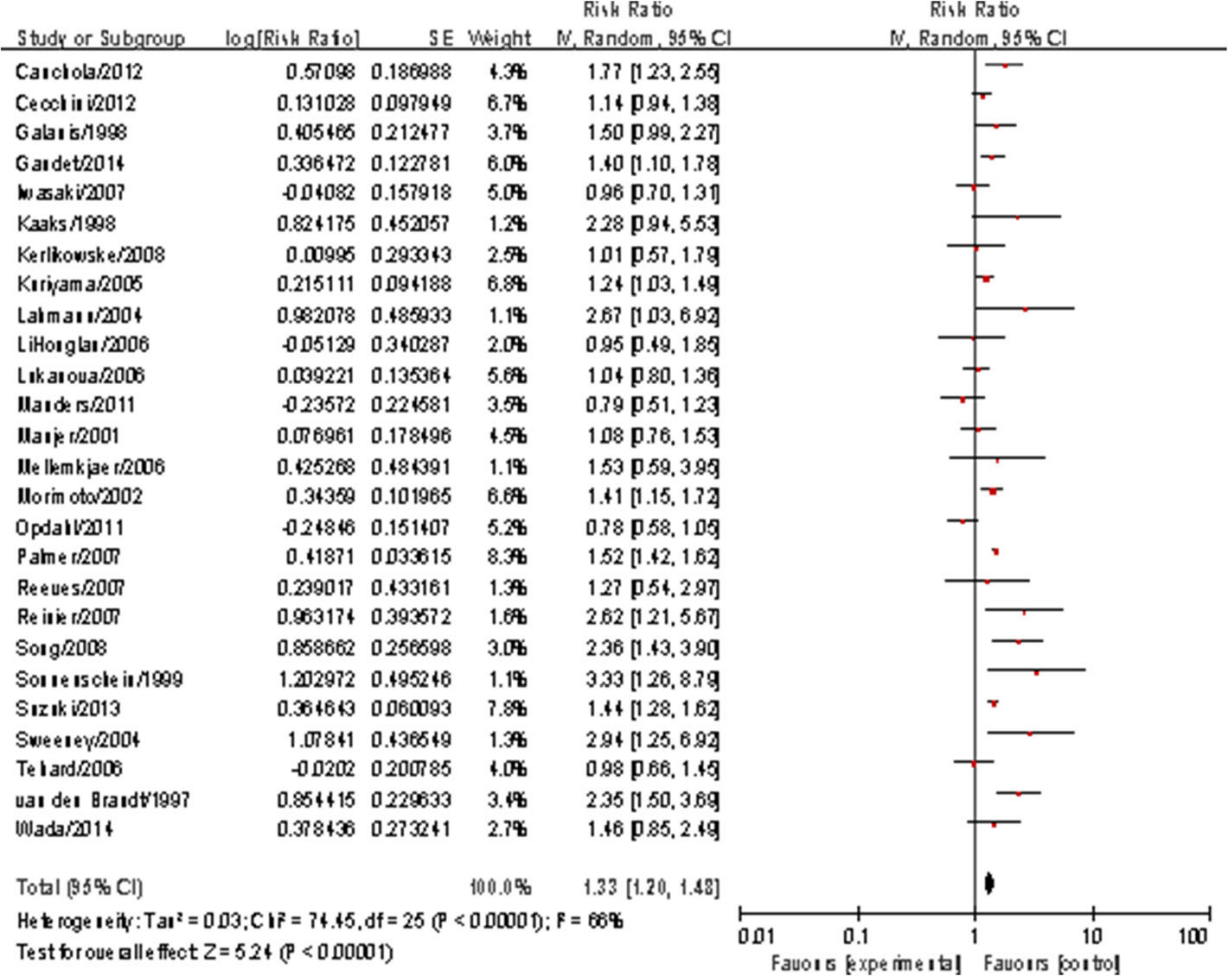
Forest plot of the body mass index (BMI) and relative risk of breast cancer in postmenopausal women (The highest vs. lowest BMI categories are being compared, the pooled RR was 1.33 (1.20–1.48), which showed a positive association between BMI and breast cancer risk in postmenopausal women)

In this study, we selected the RRs corresponding to the highest BMI categories as the highest dose, and the RRs corresponding to the normal BMI categories as the lowest dose. Eighteen studies reported on the relationship of BMI with premenopausal breast cancer risk, the summary RR was 0.94(95%CI: 0.81–1.11) (see Fig 2), and heterogeneity among these studies was statistically significant (*P* = 0.01, *I* ^2^ = 49%). Both Begg’s regression test (*P* = 0.705) and Egger’s correlation test (*P* = 0.347) showed no publication bias. Another 26 studies reported a link between BMI and breast cancer risk among postmenopausal women, with a summary RR of 1.33(95%CI: 1.20–1.48) (see Fig 3) along with evidence of heterogeneity (*P* < 0.01, *I* ^2^ = 66%). The Begg’s correlation test (*P* = 0.113) and Egger’s regression test (*P* = 0.603) showed no publication bias for these studies, as shown in Table 1.

**Table 1:** Subgroup analysis showing differences between studies included in the meta-analysis (highest versus lowest BMI)

Variables	Number of studies	Number of cases	Test of association		Test of heterogeneity		Publication bias		*P* ^a^
			Pooled RR (95% CI)	*P* value	*I* ^2^ (%)	*P* value	Begg’s *P*value	Egger’s *P* value	
All	31	43,698	1.19(1.06–1.33)	0.002	67	<0.00001	0.696	0.542	
Menopausal status									0.0006
Pre-^b^	18	5588	0.94(0.81–1.11)	0.49	49	0.01	0.705	0.347	
Post-^c^	26	35,012	1.33(1.20–1.48)	<0.0001	66	<0.00001	0.113	0.603	
Post- age									0.400
above 65	4	3580	1.53(1.39–1.68)	<0.0001	0	0.68	0.734	0.581	
below 65	4	1346	1.35(1.04–1.76)	0.02	55	0.09	0.308	0.208	
Post- ER status									<0.0001
ER + ^d^	6	7776	1.32(1.23–1.42)	<0.0001	0	0.67	1.000	0.679	
ER-^e^	5	1109	0.87(0.72–1.05)	0.15	0	0.57	0.806	0.496	
Post- HRT use									0.06
Never	8	8684	1.43(1.21–1.68)	<0.0001	55	0.03	0.902	0.811	
Ever	4	2580	0.94(0.63–1.40)	0.76	61	0.05	0.734	0.669	
Post- Area									0.0006
America	12	20,036	1.29(1.14–1.46)	<0.0001	63	0.002	0.945	0.912	
Asia	5	2234	2.10(1.64–2.69)	<0.0001	0	0.8	0.806	0.152	
Europe	9	12,742	1.19(0.98–1.43)	0.07	71	0.0006	0.917	0.046	
Post- follow up years									0.610
Below 5	5	6482	1.21(0.97–1.51)	0.08	0	0.56	0.806	0.849	
Above 5	21	28,730	1.29(1.19–1.41)	<0.0001	59	0.0003	0.415	0.792	
Pre- follow up years									0.510
Below 5	3	1071	0.82(0.60–1.12)	0.22	33	0.23	1.000	0.190	
Above 5	15	4517	0.92 (0.79–1.08)	0.32	41	0.05	0.921	0.202	
Pre- Area									0.004
America	5	1322	1.10(0.77–1.57)	0.61	71	0.008	0.858	0.304	
Asia	5	1179	1.23(0.95–1.59)	0.12	0	0.44	0.806	0.698	
Europe	8	3087	0.79(0.70–0.88)	<0.0001	0	0.49	0.283	0.387	

^a^ indicates that P value was used for comparing the differences among subgroups. ^b^ indicates premenopausal women, c indicates postmenopausal women, d indicates estrogen receptor positive status, e indicates estrogen receptor negative status

### Subgroup analyses

The subgroup analyses are shown in Table 1. There was a significant positive association in the postmenopausal women with no prior history of HRT use (RR = 1.43, 95% CI: 1.21–1.68) and those women with estrogen receptor positive status (RR = 1.32, 95% CI: 1.23–1.42). However, no association was observed in the group of postmenopausal women with a previous history of HRT use (RR = 0.94, 95% CI: 0.63–1.40) or among women with an estrogen receptor negative status (RR = 0.87, 95% CI: 0.72–1.05). The subgroup analysis between those that were below 65 years old (RR = 1.35, 95% CI: 1.04–1.76) and those above 65 (RR = 1.53, 95% CI: 1.39–1.68) both showed a positive association between BMI and breast cancer in postmenopausal women, and the heterogeneity among studies with an age above 65 was not statistically significant (*P* = 0.68, *I* ^2^ = 0). When subgroup analysis was done for different geographical locations, it showed significant associations between BMI and breast cancer in Asia (RR = 2.10, 95%CI: 1.64–2.69) and America (RR = 1.29, 95%CI: 1.14–1.46), but poor association in Europe (RR = 1.19, 95%CI: 0.98–1.43). Interestingly, for premenopausal women, an inverse significant association between BMI and breast cancer was detected in Europe (RR = 0.79, 95%CI: 0.70–0.88), but no significant associations in America (RR = 1.10, 95%CI: 0.77–1.57) or Asia (RR = 1.23, 95% CI: 0.95–1.59).

Additionally, we conducted a subgroup analysis stratified by follow-up year, the number of cases in the studies with shorter follow-up is lower, and consequently the power is reduced. In postmenopausal women, there was a significant association between BMI and breast cancer risk when the follow-up was above 5 years(RR = 1.29, 95%CI: 1.19–1.41), while no significant association was detected when the follow-up was below 5 years(RR = 1.21, 95%CI: 0.97–1.51). In premenopausal, there was no significant association between BMI and breast cancer risk in both subgroups.

### Dose-response analyses

**Fig. 4:**
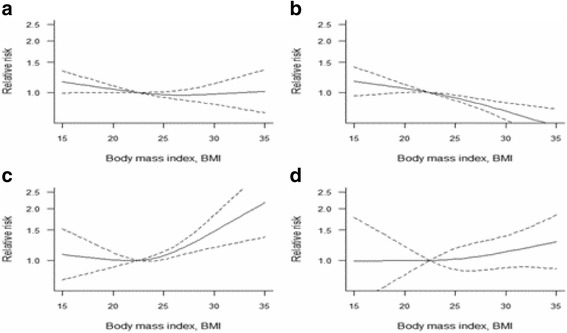
Non-linear dose-response analysis of body mass index (BMI) and relative risk of breast cancer in premenopausal women. (The solid line and the dash line represent the estimated RR and its 95%CI. **a**: All premenopausal women, **b**: European women; **c**: Asian women; **d**: American women)

**Fig. 5:**
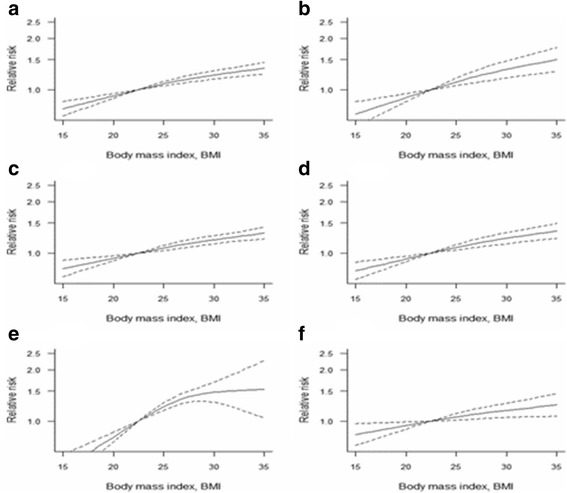
Non-linear dose-response analysis of body mass index (BMI) and relative risk of breast cancer in postmenopausal women. (The solid line and the dash line represent the estimated RR and its 95%CI. **a**: All postmenopausal women, **b**: no previous history of HRT use, **c**: estrogen receptor-positive status, **d**: American women, **e**: Asian women, **f**: European women)

For postmenopausal women, 26 studies including 62 comparisons reported RRs for corresponding BMI categories. The dose-response meta-analysis indicated a positive non-linear association between BMI and breast cancer risk among postmenopausal women. When compared to the mean level of the normal BMI range, it showed a 3.4% increased breast cancer risk per 1 kg/m^2^. In terms of premenopausal women, a dose-response analysis including 18 studies with 25 comparisons was conducted, the non-liner relationship between BMI and breast cancer risk showed no statistical significance among total premenopausal women (*χ* [2]=3.574, *P* = 0.168). In the subgroup analysis of premenopausal women, high BMI showed a decreased breast cancer risk in European women, and it showed a 2.3% decreased breast cancer risk per 1 kg/m^2^ compared to 21.5 kg/m^2^. The detailed information of the dose-response meta-analysis results and charts of premenopausal and postmenopausal women as well as the subgroup analyses are shown in Table 2, Fig. 4 and Fig. 5.

**Table 2:** The results of non-liner relationship between BMI and breast cancer risk

	21.5 kg/m^2^	22.5 kg/m^2^	23.5 kg/m^2^	25.0 kg/m^2^	26.5 kg/m^2^	30.0 kg/m^2^
Post	1(Reference)	1.03(1.02–1.05)	1.07(1.04–1.10)	1.12(1.08–1.17)	1.17(1.11–1.23)	1.26(1.18–1.35)
Post-America	1(Reference)	1.03(1.02–1.05)	1.07(1.03–1.10)	1.11(1.06–1.17)	1.16(1.09–1.24)	1.26(1.15–1.38)
Post-Europe	1(Reference)	1.02(1.00–1.04)	1.04(1.01–1.09)	1.08(1.02–1.15)	1.12(1.03–1.21)	1.19(1.06–1.33)
Post-Asia	1(Reference)	1.10(1.07–1.13)	1.19(1.13–1.25)	1.32(1.24–1.41)	1.42(1.31–1.54)	1.60(1.37–1.87)
Post-HRT^a^ non user	1(Reference)	1.04(1.02–1.07)	1.09(1.04–1.14)	1.16(1.08–1.25)	1.23(1.12–1.35)	1.37(1.20–1.57)
Post-ER + ^b^	1(Reference)	1.03(1.01–1.04)	1.06(1.03–1.09)	1.10(1.05–1.15)	1.14(1.08–1.22)	1.23(1.15–1.32)
Pre-Europe	1(Reference)	0.98(0.96–1.00)	0.95(0.92–0.99)	0.91(0.86–0.96)	0.87(0.81–0.93)	0.76(0.67–0.86)
Pre-Asia	1(Reference)	1.00(0.97–1.04)	1.02(0.96–1.08)	1.07(0.99–1.16)	1.16(1.05–1.27)	1.48(1.20–1.83)

The dose 21.5 kg/m^2^ is the reference group

^a^ indicates hormone replacement therapy, ^b^ indicates estrogen receptor positive status

### Publication bias

No publication bias was observed for any of the studies or the subgroup studies, except for studies on postmenopausal women in Europe using the Egger’s test (*P* = 0.046). After using a trim-and-fill analysis, no study showed bias, and the results were unchanged.

### Sensitivity analysis

For the sensitivity analysis, we omitted one study at a time in turn to assess the potential studies which may influence the main results. In the studies for premenopausal women, the summary RRs showed little variation ranging from 0.89(95%CI: 0.77–1.03) to 0.98(95%CI: 0.84–1.15), and the result was not influenced by any single study. Similarly, the sensitivity analysis in postmenopausal women showed stable results, which ranged from 1.30 (95%CI: 1.17–1.45) to 1.36 (95%CI: 1.23–1.51).

## Discussions

In this meta-analysis, there was a significant positive association between BMI and breast cancer risk among postmenopausal women but no correlation among total premenopausal women. Non-linear dose-response analysis showed that the breast cancer risk increased by 3.4% for every 1 kg/m^2^ increment of BMI in postmenopausal women.

Heterogeneity was observed among the studies included in our meta-analysis. The diversity most likely comes from differences in study design, sample source and size, reference categories, different BMI cut-offs, different weight distribution, length of follow up, racial difference, ER status, HRT use or other potential confounders. We conducted a subgroup analysis to explore the heterogeneity and potential factors influencing the associations. The heterogeneity was zero (*I* ^2^ = 0) when stratified by ER status, suggesting ER status may be an interactive factor for the relationship between BMI and breast cancer risk. The lastest WCRF 2017 report showed that in postmenopausal women BMI was significantly positively associated with ER+ breast cancer, but not ER– breast cancers, it was consistent with our study.

The mechanism of the association between obesity and breast cancer remains unclear. Currently it is thought that adipose tissue of obese women produces excessive estrogen hormone, which in turn stimulates more estrogen-sensitive breast tissues that may already have a propensity for hyper-stimulation, ultimately promoting the formation and development of tumors [46]. In premenopausal women, estrogen is mainly derived from the ovaries, while after menopause, most circulating estrogen is derived from the conversion of adrenal androgens by means of adipose aromatase [47, 48]. Therefore, women with higher amounts of body fat tend to have higher levels of circulating estrogen. Moreover, studies have found a stronger relationship between obesity and estrogen receptor (ER) positive breast cancers than between obesity and ER-negative cancers [42, 44]. Interestingly, a history of using hormone therapy attenuates the relationship between obesity and breast cancer risk among postmenopausal women [3, 4]. These findings provide further evidence for the estrogen availability theory among postmenopausal women, and our analysis also confirmed this point. In addition, other hormonal factors including insulin resistance, adipocytokines, AMP-activated protein kinase and leptin, which are related to obesity, are also important factors for the formation and development of breast cancer [47, 49–51].

Though hormonal etiology may partially explain the differential role of BMI in relation to breast cancer risk, it is increasingly considered that premenopausal breast cancer cases might have distinct etiologies from postmenopausal breast cancer cases. A recent study [52] showed that obesity has divergent impacts on risk of aggressive subtypes of breast cancer in premenopausal versus postmenopausal women, and that some non-hormonal pathways may also mediate the association between obesity and TN breast cancers among premenopausal women. Obesity-related factors such as inflammation, elevated levels of insulin and insulin-like growth factors, or other carcinogens may play different roles on postmenopausal and premenopausal breast cancer risk.

The heterogeneity observed in this meta-analysis remained after stratification by a previous history of HRT use or not, and the positive association only existed in postmenopausal women who never used HRT. The results showed that HRT may be a factor influencing the relationship between BMI and postmenopausal breast cancer, and it is similar to the WCRF 2017 report. One study reported that the relationship between HRT and breast cancer risk was related to race, BMI and breast density among postmenopausal women [53]. Compared with HRT non-users, HRT users were at increased risk of breast cancer; however, there was no statistical correlation between HRT and breast cancer when the women were obese. Considering the above results, it is likely that BMI and a history of HRT use may interact to affect the incidence of breast cancer.

When stratified by geographical location among postmenopausal women, a significant non-linear dose-response relationship between BMI and breast cancer risk existed in American and Asian women, but not among European women. These results were inconsistent with the previous report conducted by Xia Xiaoping, et al. [54] Xia showed that significant non-linear dose-response association of BMI and BC risk was identified in White women, while in an Asian subgroup, no significant association was observed. This may be because the studies included in our study were divided into America, Europe and Asia, whereas in their study ethnicity was divided into White and Asian and did not take into account Black or African Americans, which make up a significant proportion of the US population. In our study, the breast cancer risk in Asia was considerably higher than in America, this may be because Asian people are more likely to have higher body fat with less lean mass and skeletal muscle [55], which may increase breast cancer risk. In China, changes in reproductive patterns (such as family planning, less breastfeeding, or older age at first full-term pregnancy) are affecting the concentrations of sex hormones, and coupled with changing lifestyle and dietary factors may be related to an increasing risk of breast cancer [56]. For Europe, a larger heterogeneity (*I* ^2^ = 71%) between studies remained, and the relationship between BMI and BC risk was not statistically significant. In WCRF 2017 report, dose-response meta-analyses for postmenopausal breast cancer by geographical location showed a statistically significant increased risk in North American and European studies, and a stronger association in Asian studies. The AICR&WCRF 2017 report includes 19 cohort and case-control studies of Europe (*I* ^2^ = 75%), while 9 cohort studies of Europe (*I* ^2^ = 71%) were included in this study. Potential confounding factors such as the difference in fat distribution, life styles or a possible interaction effect might be the source of the high heterogeneity. It necessitates further studies in order to provide more insights into such effects among European women.

For premenopausal women, the AICR&WCRF report summarized the epidemiologic evidence available up to 2017 and concluded that there is a substantial dose response relationship for the inverse association between BMI and risk of breast cancer among premenopausal women. However, the non-significant association of BMI with risk of premenopausal breast cancer observed in this study is not consistent with the WCRF report. This may be because the studies included in this study differed from those included in AICR&WCRF 2017 report. The studies included in this article were all cohort studies which were more convincing, however, the number of studies included in AICR&WCRF 2017 report were more than this study, and more recent studies may be included. Therefore, the sample source and size, different fat distribution, follow-up year, race, ER status, HRT use or other confounders are possibly the reasons of differences.

A lack of data prohibited many subgroup analyses from being carried out. However, stratification by geographical location demonstrated an inverse significant association between BMI and breast cancer among European premenopausal women but not among Asian and American premenopausal women. This conclusion is consistent with the AICR&WCRF 2017 report when stratified by geographical location. When compared to women with a normal BMI (normal BMI was set as 21.5), women with a higher BMI were at a decreased risk of breast cancer risk by 2.3%. In Asian women, the results showed a positive non-linear relationship between BMI and breast cancer when BMI was above 25 kg/m^2^. In American premenopausal women, there was no association between BMI and breast cancer. These results are comparable to those observed in a systematic review by Amadou et al. [57], which explored the relationship between overweight, obesity and premenopausal breast cancer according to ethnicity. However, the results in this review are not completely consistent with our findings in premenopausal women, a significant inverse association remained among Africans and Caucasians after stratification, while a significant positive association was detected among Asian women. This is possibly due to the inclusion of 19 case control studies and 11 cohort studies, while all 18 studies within our meta-analysis were cohort. The similarities were the results of Europeans in our analysis and the Caucasians in the study by Amadou et al. [57], in addition, Asian women in our study showed a positive correlation when BMI was above 25 kg/m^2^_,_ which was similar to the report of Amadou et al. These results suggest that ethnicity or geographical location may be a factor influencing the relationship between BMI and breast cancer risk, but this conclusion is speculative. In this meta-analysis, breast cancer risk could be influenced by overweight or obesity according to different geographical locations, irrespective of menopausal status. The difference may be related to different genetic susceptibility among different ethnic groups, as demonstrated for breast cancer susceptibility genes (BRCA1 or BRCA2) mutations previously [58].

BMI, adult weight gain, body weight and fat distribution may play a role in the prognosis of breast cancer separately or together in combination. IARC working group [59, 60] reported that higher weight gain was associated with decreased risk for premenopausal women and increased risk for postmenopausal, and the WCRF also reported that adult weight gain is a probable cause of postmenopausal breast cancer. Weight gain is a widely used index of obesity in adult, and a dynamic measure unlike static measures such as BMI [61], therefore, it may be better to combine BMI and adult weight gain to explore the relationship between obesity and breast cancer.

The studies included in this meta-analysis were all cohort studies, with large sample sizes, and adequate subgroup analyses. Moreover, the confirmation of the breast cancer cases was done by diagnosis, hospital medical record or linkage data. Thus, the results were persuasive, and were less likely to suffer from recall bias and selection bias. However, some limitations also exist for this meta-analysis; the measurement of BMI was determined mostly by questionnaire investigation or self-reported, and people tended to report their weight lower and height higher than reality. The adjustments factors of each study were not the same, thus the RRs we extracted from the studies were possibly affected by potential confounders even after selecting the adjusted RRs. In addition, the categorization of BMI in a number of articles included in this study was not in accordance with the WHO standard, thus misclassifications may be caused when we conduct the highest versus the lowest BMI meta-analysis.

## Conclusion

In conclusion, BMI had different effects on premenopausal and postmenopausal breast cancer risks. In postmenopausal women, breast cancer risk increased along with increasing BMI. In premenopausal women, there was no signification between BMI and breast cancer. This is inconsistent with the 2017 report of AICR&WCRF which showed higher BMI is defined as probably decreasing breast cancer risk. However, most conclusions stratified by various factors in this study are consistent with AICR&WCRF.

Subgroup analyses suggested that the geographical location or genetic factors may influence the relationship between BMI and breast cancer. Further research is needed to provide more continuous updating insights.

## Additional files

Additional file 1: Study characteristics of published cohort studies on body mass index(BMI) and breast cancer risk. (DOC 725 kb)

Additional file 2: The related data and materials in this study. (ZIP 1785 kb)

## Abbreviations

BMI: Body Mass Index
BRCA: Breast Cancer Susceptibility Genes
CI: Confidence Interval
CNKI: China National Knowledge Infrastructure
ER: Estrogen Receptor
HR: Hazard Ratio
HRT: Hormone Replacement Therapy
IARC: American Institute for Cancer Research
NOS: Newcastle-Ottawa Scale
PR: Progesterone Receptor
RR: Relative Risk
VIP: Chinese Scientific Journals
WCRF: World Cancer Research Fund
