# Supplementary material for: Body mass index had different effects on premenopausal and postmenopausal breast cancer risks: a dose-response meta-analysis with 3,318,796 subjects from 31 cohort studies
Source: BMC Public Health. 2017 Dec 8;17:936. doi: 10.1186/s12889-017-4953-9 (PMC5721381; doi:10.1186/s12889-017-4953-9)
Supplement: Supplementary file 1 — Study characteristics of published cohort studies on body mass index(BMI) and breast cancer risk. (DOC 725 kb) [file 12889_2017_4953_MOESM1_ESM.doc]

**Table 1 Study characteristics of published cohort studies on body mass index(BMI) and breast cancer risk**

| ID | Author, year | Type | Country  (Area) | Follow-up | Cases | N | Study time | Age at baseline | NOS | Group | BMI | RR^i^(95%CI^j^) | Adjusting factors |
| --- | --- | --- | --- | --- | --- | --- | --- | --- | --- | --- | --- | --- | --- |
| 1 | Li Honglan, 2006 | Ir | China  (Asia) | 5.66 | 432 | 73410 | 1997-2000 | 40-70 | 8 | Pre-^a^ | <22.5 | 1.00(Reference) | age,education, menstrual status, pregnancy,age at menopausal, age at first birth,family history of breast cancer |
|  |  |  |  |  |  |  |  |  |  |  | 22.5-25.20 | 1.06(0.78-1.45) |  |
|  |  |  |  |  |  |  |  |  |  |  | ≥25.21 | 1.04(0.73-1.49) |  |
|  |  |  |  |  |  |  |  |  |  | Post-^b^ | <22.5 | 1.00(Reference) |  |
|  |  |  |  |  |  |  |  |  |  |  | 22.5-25.20 | 1.14(0.76-1.70) |  |
|  |  |  |  |  |  |  |  |  |  |  | ≥25.21 | 1.77(1.23-2.56) |  |
| 2 | Cecchini,2012 | Ir | North America  (America) | 4.1 years for P-1 and 6.4 years for star | 810 | 31731 | 1992-1998 | ≥35 | 7 | P-1 Pre- | <25 | 1.00(Reference) | treatment,Gail score, age, history of diabetes, history of oral contraceptive use, history of estrogen use, years of cigarette smoking |
|  |  |  |  |  |  |  |  |  |  |  | 25-29 | 1.59(1.05-2.42) |  |
|  |  |  |  |  |  |  |  |  |  |  | ≥30 | 1.7(1.10-2.63) |  |
|  |  |  |  |  |  |  |  |  |  | star post- | <25 | 1.00(Reference) |  |
|  |  |  |  |  |  |  |  |  |  |  | 25-29 | 1.04(0.85-1.29) |  |
|  |  |  |  |  |  |  |  |  |  |  | ≥30 | 1.16(0.94-1.42) |  |
|  |  |  |  |  |  |  |  |  |  | P-1 post- | <25 | 1.00(Reference) |  |
|  |  |  |  |  |  |  |  |  |  |  | 25-29 | 1.22(0.81-1.85) |  |
|  |  |  |  |  |  |  |  |  |  |  | ≥30 | 1.09(0.70-1.69) |  |
|  |  |  |  |  |  |  |  |  |  | star/P-1 post- | <25 | 1.00(Reference) |  |
|  |  |  |  |  |  |  |  |  |  |  | 25-29 | 1.07(0.88-1.30) |  |
|  |  |  |  |  |  |  |  |  |  |  | ≥30 | 1.14(0.94-1.38) |  |
|  |  |  |  |  |  |  |  |  |  | star/P-1 post- AND ER+^c^ | <25 | 1.00(Reference) |  |
|  |  |  |  |  |  |  |  |  |  |  | 25-29 | 1.14(0.91-1.43) |  |
|  |  |  |  |  |  |  |  |  |  |  | ≥30 | 1.23(0.98-1.55) |  |
|  |  |  |  |  |  |  |  |  |  | star/P-1 post- AND ER-^d^ | <25 | 1.00(Reference) |  |
|  |  |  |  |  |  |  |  |  |  |  | 25-29 | 1.00(0.68-1.48) |  |
|  |  |  |  |  |  |  |  |  |  |  | ≥30 | 1.03(0.70-1.52) |  |
|  |  |  |  |  |  |  |  |  |  | P-1 Pre- AND ER+ | <25 | 1.00(Reference) |  |
|  |  |  |  |  |  |  |  |  |  |  | 25-29 | 1.41(0.82-2.43) |  |
|  |  |  |  |  |  |  |  |  |  |  | ≥30 | 1.78(1.03-3.07) |  |
|  |  |  |  |  |  |  |  |  |  | P-1 Pre- AND ER- | <25 | 1.00(Reference) |  |
|  |  |  |  |  |  |  |  |  |  |  | 25-29 | 2.52(1.19-5.33) |  |
|  |  |  |  |  |  |  |  |  |  |  | ≥30 | 1.79(0.76-4.22) |  |
| 3 | Galanis,1998 | ci | American  (America) | 14.9 | 378 | 17628 | 1975-1980 | NA | 8 | Pre- | <19.6 | 1.00(Reference) | age,years ofeducation,ethnicity(Asian,Hawaiian,or Caucasian),drinking status |
|  |  |  |  |  |  |  |  |  |  |  | 19.6-21.0 | 1.30(0.60-2.60) |  |
|  |  |  |  |  |  |  |  |  |  |  | 21.1-23.0 | 1.60(0.80-3.20) |  |
|  |  |  |  |  |  |  |  |  |  |  | 23.1-26 | 1.01(0.50-2.20) |  |
|  |  |  |  |  |  |  |  |  |  |  | >26 | 1.9(0.90-3.90) |  |
|  |  |  |  |  |  |  |  |  |  | Post- | <19.6 | 1.00(Reference) |  |
|  |  |  |  |  |  |  |  |  |  |  | 19.6-21.0 | 1.10(0.70-1.60) |  |
|  |  |  |  |  |  |  |  |  |  |  | 21.1-23.0 | 1.11(0.70-1.70) |  |
|  |  |  |  |  |  |  |  |  |  |  | 23.1-26 | 1.30(0.90-2.00) |  |
|  |  |  |  |  |  |  |  |  |  |  | >26 | 1.50(1.01-2.30) |  |
|  |  |  |  |  |  |  |  |  |  | All^e^ | <19.6 | 1.00(Reference) |  |
|  |  |  |  |  |  |  |  |  |  |  | 19.6-21.0 | 1.20(0.80-1.70) |  |
|  |  |  |  |  |  |  |  |  |  |  | 21.1-23.0 | 1.30(0.90-1.90) |  |
|  |  |  |  |  |  |  |  |  |  |  | 23.1-26 | 1.40(1.01-2.00) |  |
|  |  |  |  |  |  |  |  |  |  |  | >26 | 1.80(1.30-2.60) |  |
|  |  |  |  |  |  |  |  |  |  | post- age≤65 | <21.1 | 1.00(Reference) |  |
|  |  |  |  |  |  |  |  |  |  |  | 21.1-24 | 0.80(0.50-1.30) |  |
|  |  |  |  |  |  |  |  |  |  |  | ≥24 | 1.30(0.90-2.02) |  |
|  |  |  |  |  |  |  |  |  |  | post- age≥65 | <21.1 | 1.00(Reference) |  |
|  |  |  |  |  |  |  |  |  |  |  | 21.1-24 | 1.40(0.90-2.10) |  |
|  |  |  |  |  |  |  |  |  |  |  | ≥24 | 1.60(1.10-2.50) |  |
| 4 | Gaudet,2014 | ir | US  (America) | 11.58 | 1088 | 28965 | 1982-1992 | NA | 8 | post- | <25 | 1.00(Reference) | height,education,parity,age at first birth,smoking,alcohol,race,family history of breast cancer,aral contraceptive use,diabetes,age at menopause,exercise,benign breast disease,recent mammography screening,hormone use，waist circumference |
|  |  |  |  |  |  |  |  |  |  |  | 25-29.9 | 1.26(1.07-1.48) |  |
|  |  |  |  |  |  |  |  |  |  |  | ≥30 | 1.40(1.10-1.78) |  |
|  |  |  |  |  |  |  |  |  |  | post- AND ER+ | <25 | 1.00(Reference) |  |
|  |  |  |  |  |  |  |  |  |  |  | 25-29.9 | 1.28(1.06-1.54) |  |
|  |  |  |  |  |  |  |  |  |  |  | ≥30 | 1.41(1.08-1.85) |  |
|  |  |  |  |  |  |  |  |  |  | post- AND ER- | <25 | 1.00(Reference) |  |
|  |  |  |  |  |  |  |  |  |  |  | 25-29.9 | 0.96(0.65-1.43) |  |
|  |  |  |  |  |  |  |  |  |  |  | ≥30 | 0.61(0.35-1.08) |  |
| 5 | Canchola,2012 | ir | American  (America) | 12.1 | 2321 | 52642 | NA | 56-70 | 7 | Post- AND ER+PR+^f^ | <25 | 1.00(Reference) | height,age at menarche,parity,age at first full-term pregnancy,history of benign breast boisy,family history of breast cancer,alcohol,use of HT |
|  |  |  |  |  |  |  |  |  |  |  | 25-29 | 1.13(1.00-1.28) |  |
|  |  |  |  |  |  |  |  |  |  |  | ≥30 | 1.20(1.03-1.40) |  |
|  |  |  |  |  |  |  |  |  |  | Post- AND ER+PR-^g^ | <25 | 1.00(Reference) |  |
|  |  |  |  |  |  |  |  |  |  |  | 25-29 | 0.90(0.69-1.18) |  |
|  |  |  |  |  |  |  |  |  |  |  | ≥30 | 0.84(0.58-1.21) |  |
|  |  |  |  |  |  |  |  |  |  | Post- AND ER-PR- | <25 | 1.00(Reference) |  |
|  |  |  |  |  |  |  |  |  |  |  | 25-29 | 1.13(0.87-1.47) |  |
|  |  |  |  |  |  |  |  |  |  |  | ≥30 | 0.77(0.53-1.12) |  |
| 6 | Iwasaki,2007 | ir | Japan  (Asia) | 9.9 | 441 | 55537 | 1990-2002 | 40-69 | 9 | Pre- | <19 | 1.00(Reference) | age,area,number of births,age at first birth,height |
|  |  |  |  |  |  |  |  |  |  |  | 19-20.9 | 0.94(0.49-1.80) |  |
|  |  |  |  |  |  |  |  |  |  |  | 21-22.9 | 0.89(0.47-1.68) |  |
|  |  |  |  |  |  |  |  |  |  |  | 23-24.9 | 0.95(0.50-1.81) |  |
|  |  |  |  |  |  |  |  |  |  |  | 25-26.9 | 1.07(0.53-2.13) |  |
|  |  |  |  |  |  |  |  |  |  |  | 27-29.9 | 1.47(0.72-3.02) |  |
|  |  |  |  |  |  |  |  |  |  |  | ≥30 | 1.35(0.53-3.47) |  |
|  |  |  |  |  |  |  |  |  |  | Post- | <19 | 1.00(Reference) |  |
|  |  |  |  |  |  |  |  |  |  |  | 19-20.9 | 1.39(0.66-2.92) |  |
|  |  |  |  |  |  |  |  |  |  |  | 21-22.9 | 1.43(0.71-2.91) |  |
|  |  |  |  |  |  |  |  |  |  |  | 23-24.9 | 1.63(0.81-3.31) |  |
|  |  |  |  |  |  |  |  |  |  |  | 25-26.9 | 1.49(0.71-3.11) |  |
|  |  |  |  |  |  |  |  |  |  |  | 27-29.9 | 1.56(0.72-3.38) |  |
|  |  |  |  |  |  |  |  |  |  |  | ≥30 | 2.28(0.94-5.53) |  |
| 7 | Kaaks,1998 | ir | Netherlands  (Europe) | 10.6 | 275 | 11663 | 1984-1996 | NA | 8 | Pre- | ≤22.5 | 1.00(Reference) | age, ageat menarche, age at first childbirth, age at menopause, and number ofliveborn children , and menopausal status |
|  |  |  |  |  |  |  |  |  |  |  | 22.5-24.54 | 1.08(0.71-1.64) |  |
|  |  |  |  |  |  |  |  |  |  |  | 24.54-27.15 | 0.76(0.47-1.24) |  |
|  |  |  |  |  |  |  |  |  |  |  | >27.15 | 1.04(0.65-1.68) |  |
|  |  |  |  |  |  |  |  |  |  | Post- natural menopause | ≤22.5 | 1.00(Reference) |  |
|  |  |  |  |  |  |  |  |  |  |  | 22.5-24.54 | 0.69(0.34-1.40) |  |
|  |  |  |  |  |  |  |  |  |  |  | 24.54-27.15 | 0.49(0.24-1.00) |  |
|  |  |  |  |  |  |  |  |  |  |  | >27.15 | 0.81(0.43-1.51) |  |
|  |  |  |  |  |  |  |  |  |  | Post- hysterectomy or ovariectomy | ≤22.5 | 1.00(Reference) |  |
|  |  |  |  |  |  |  |  |  |  |  | 22.5-24.54 | 1.47(0.58-3.70) |  |
|  |  |  |  |  |  |  |  |  |  |  | 24.54-27.15 | 1.57(0.63-3.92) |  |
|  |  |  |  |  |  |  |  |  |  |  | >27.15 | 1.5(0.61-3.70) |  |
| 8 | Kerlikowske,2008 | ir | American  (America) | NA | 4446 | 287155 | 1996-2005 | ≥40 | 7 | post- AND HRT^h^ never use | <18.5 | 1.03(0.82-1.30) | race.age,mammography use or first screening examination,registry |
|  |  |  |  |  |  |  |  |  |  |  | 18.5-24.9 | 1.00(Reference) |  |
|  |  |  |  |  |  |  |  |  |  |  | 25-29.9 | 1.12(1.05-1.21) |  |
|  |  |  |  |  |  |  |  |  |  |  | 30-34.9 | 1.20(1.10-1.31) |  |
|  |  |  |  |  |  |  |  |  |  |  | ≥35 | 1.30(1.17-1.45) |  |
|  |  |  |  |  |  |  |  |  |  | post- AND ER+ | <18.5 | 1.10(0.82-1.49) |  |
|  |  |  |  |  |  |  |  |  |  |  | 18.5-24.9 | 1.00(Reference) |  |
|  |  |  |  |  |  |  |  |  |  |  | 25-29.9 | 1.17(1.07-1.29) |  |
|  |  |  |  |  |  |  |  |  |  |  | 30-34.9 | 1.25(1.11-1.41) |  |
|  |  |  |  |  |  |  |  |  |  |  | ≥35 | 1.34(1.16-1.54) |  |
|  |  |  |  |  |  |  |  |  |  | post- AND ER- | <18.5 | 0.86(0.42-1.75) |  |
|  |  |  |  |  |  |  |  |  |  |  | 18.5-24.9 | 1.00(Reference) |  |
|  |  |  |  |  |  |  |  |  |  |  | 25-29.9 | 0.96(0.78-1.18) |  |
|  |  |  |  |  |  |  |  |  |  |  | 30-34.9 | 0.98(0.76-1.27) |  |
|  |  |  |  |  |  |  |  |  |  |  | ≥35 | 0.95(0.68-1.32) |  |
| 9 | Kuriyama, 2005 | ir | Japan  (Asia) | 9 | 115 | 17353 | 1984-1992 | ≥40 | 8 | All | 18.5-24.9 | 1.00(Reference) | age.smoking,drinking,consumption of meat,fish,fruit,green or yellow vegetables,type of health insurance,menopausal status,age at menarche,age at end of first pregnancy |
|  |  |  |  |  |  |  |  |  |  |  | 25-27.4 | 1.20(0.75-1.93) |  |
|  |  |  |  |  |  |  |  |  |  |  | 27.5-29.9 | 1.55(0.84-2.87) |  |
|  |  |  |  |  |  |  |  |  |  |  | ≥30 | 1.90(0.87-4.15) |  |
|  |  |  |  |  |  |  |  |  |  | post- | 18.5-24.9 | 1.00(Reference) |  |
|  |  |  |  |  |  |  |  |  |  |  | 25-27.4 | 1.70(0.94-3.07) |  |
|  |  |  |  |  |  |  |  |  |  |  | 27.5-29.9 | 1.82(0.80-4.12) |  |
|  |  |  |  |  |  |  |  |  |  |  | ≥30 | 2.67(1.03-6.92) |  |
|  |  |  |  |  |  |  |  |  |  | pre- | 18.5-24.9 | 1.00(Reference) |  |
|  |  |  |  |  |  |  |  |  |  |  | 25-27.4 | 0.85(0.32-2.24) |  |
|  |  |  |  |  |  |  |  |  |  |  | 27.5-29.9 | 0.84(0.24-2.88) |  |
|  |  |  |  |  |  |  |  |  |  |  | ≥30 | NA |  |
| 10 | Lacey,2009 | ir | US  (America) | 5 | 2085 | 70575 | 1993-2001 | 55-74 | 8 | All | <18.5 | 0.88(0.59-1.32) | age,screening centre,age at menarche,age at menopause,family history of breast cancer,benign breast disease,height,menopausal hormone therapy,age at first birth,parity,calendar time. |
|  |  |  |  |  |  |  |  |  |  |  | 18.5-24.9 | 1.00(Reference) |  |
|  |  |  |  |  |  |  |  |  |  |  | 25-29 | 1.06(0.95-1.17) |  |
|  |  |  |  |  |  |  |  |  |  |  | 30-34.9 | 1.10(0.97-1.26) |  |
|  |  |  |  |  |  |  |  |  |  |  | ≥35 | 1.21(1.02-1.43) |  |
| 11 | Lahmann,2004 | ir | Eurpean  (Europe) | 4.7 | 1897 | 176886 | 1992-2000 | 25-70 | 8 | pre- | <21.6 | 1.00(Reference) | study centre,age,education,smoking,alcohol,parity,age at first pregnancy,age at menarche and current pill use. |
|  |  |  |  |  |  |  |  |  |  |  | 21.6-23.5 | 0.95(0.73-1.23) |  |
|  |  |  |  |  |  |  |  |  |  |  | 23.6-25.6 | 0.78(0.59-1.04) |  |
|  |  |  |  |  |  |  |  |  |  |  | 25.7-28.7 | 0.80(0.59-1.09) |  |
|  |  |  |  |  |  |  |  |  |  |  | ≥28.8 | 0.82(0.59-1.14) |  |
|  |  |  |  |  |  |  |  |  |  | Post- AND HRT never use | <25.0 | 1.00(Reference) |  |
|  |  |  |  |  |  |  |  |  |  |  | 25.0-29.9 | 1.30(1.12-1.51) |  |
|  |  |  |  |  |  |  |  |  |  |  | ≥30 | 1.31(1.08-1.59) |  |
|  |  |  |  |  |  |  |  |  |  | Post- AND HRT ever use | <25.0 | 1.00(Reference) |  |
|  |  |  |  |  |  |  |  |  |  |  | 25.0-29.9 | 0.94(0.76-1.15) |  |
|  |  |  |  |  |  |  |  |  |  |  | ≥30 | 0.66(0.45-0.98) |  |
| 12 | Lukanova, 2006 | ir | Swedish  (Europe) | 8.3 | 514 | 35362 | 1994-2003 | 30-60 | 7 | All | 18.5-24.9 | 1.00(Reference) | age, calender year,smoking |
|  |  |  |  |  |  |  |  |  |  |  | 25-29.9 | 0.89(0.73-1.08) |  |
|  |  |  |  |  |  |  |  |  |  |  | ≥30 | 1.01(0.76-1.29) |  |
|  |  |  |  |  |  |  |  |  |  | Pre- AND age<49 | 18.5-22.1 | 1.00(Reference) |  |
|  |  |  |  |  |  |  |  |  |  |  | 22.2-24.2 | 1.31(0.77-2.28) |  |
|  |  |  |  |  |  |  |  |  |  |  | 24.3-27.1 | 0.99(0.56-1.76) |  |
|  |  |  |  |  |  |  |  |  |  |  | ≥27 | 0.58(0.29-1.11) |  |
|  |  |  |  |  |  |  |  |  |  | Post- AND age≥49 | 18.5-22.1 | 1.00(Reference) |  |
|  |  |  |  |  |  |  |  |  |  |  | 22.2-24.2 | 0.99(0.76-1.30) |  |
|  |  |  |  |  |  |  |  |  |  |  | 24.3-27.1 | 0.90(0.68-1.18) |  |
|  |  |  |  |  |  |  |  |  |  |  | ≥27 | 1.04(0.80-1.36) |  |
| 13 | Lundqvist, 2007 | ir | Swedish and Finland  (Europe) | older 22 years and younger 28.4 years | 756 older 881 younger | 14058 older 22432 younger | 1969-2002 | NA | 7 | All older subjects | <18.5 | 0.90(0.50-1.50) | age,country,smoking,physical activity,education,diabetes,parity. |
|  |  |  |  |  |  |  |  |  |  |  | 18.5-25.0 | 1.00(Reference) |  |
|  |  |  |  |  |  |  |  |  |  |  | 25.0-30 | 1.20(1.01-1.40) |  |
|  |  |  |  |  |  |  |  |  |  |  | ≥30 | 1.30(1.02-1.70) |  |
|  |  |  |  |  |  |  |  |  |  | All younger subjects | <18.5 | 0.90(0.70-1.30) |  |
|  |  |  |  |  |  |  |  |  |  |  | 18.5-25.0 | 1.00(Reference) |  |
|  |  |  |  |  |  |  |  |  |  |  | 25.0-30 | 1.10(0.90-1.30) |  |
|  |  |  |  |  |  |  |  |  |  |  | ≥30 | 0.80(0.40-1.30) |  |
| 14 | Manders,2011 | ir | Netherlands  (Europe) | 6.9 | 218 | 719 | 1999-2007 | NA | 6 | Pre- | ≤18.49 | 0.41(0.09-1.85) | parity,type of menopause,HRT use,physical activity |
|  |  |  |  |  |  |  |  |  |  |  | 18.50-22.49 | 1.00(Reference) |  |
|  |  |  |  |  |  |  |  |  |  |  | 22.50-24.99 | 0.87(0.53-1.42) |  |
|  |  |  |  |  |  |  |  |  |  |  | ≥25 | 0.75(0.43-1.31) |  |
|  |  |  |  |  |  |  |  |  |  | Post- | ＜25 | 1.00(Reference) |  |
|  |  |  |  |  |  |  |  |  |  |  | ≥25 | 1.46(0.86-2.51) |  |
| 15 | Manjer,2001 | ir | Netherlands  (Eurooe) | 13.1 | 269 | 9738 | 1977-1992 | 42-58 | 8 | Pre- | ≤20.61 | 1.00(Reference) | age,age at menarche,nulliparity,current oral contraceptive use,currert HRT,smoking,alcohol consumption,height. |
|  |  |  |  |  |  |  |  |  |  |  | 20.61-22.75 | 0.81(0.47-1.37) |  |
|  |  |  |  |  |  |  |  |  |  |  | 22.75-25.46 | 0.94(0.55-1.61) |  |
|  |  |  |  |  |  |  |  |  |  |  | ＞25.46 | 1.01(0.57-1.75) |  |
|  |  |  |  |  |  |  |  |  |  | Post- | ≤20.61 | 1.00(Reference) |  |
|  |  |  |  |  |  |  |  |  |  |  | 20.61-22.75 | 0.85(0.54-1.32) |  |
|  |  |  |  |  |  |  |  |  |  |  | 22.75-25.46 | 1.29(0.83-2.00) |  |
|  |  |  |  |  |  |  |  |  |  |  | ＞25.46 | 0.79(0.51-1.23) |  |
| 16 | Mellemkjaer,2006 | ir | Danish  (Europe) | 6.7 | 633 | 23788 | 1993-2002 | 50-64 | 9 | Post- AND HRT Never use | <18.5 | NA | parity,age at birth of first child,beign breast tumor,education,alcohol. |
|  |  |  |  |  |  |  |  |  |  |  | 18.5-25 | 1.00(Reference) |  |
|  |  |  |  |  |  |  |  |  |  |  | 25-30 | 1.34(1.01-1.80) |  |
|  |  |  |  |  |  |  |  |  |  |  | ≥30 | 1.17(0.79-1.73) |  |
|  |  |  |  |  |  |  |  |  |  | Post- AND HRT Ever use | <18.5 | 1.23(0.58-2.63) |  |
|  |  |  |  |  |  |  |  |  |  |  | 18.5-25 | 1.00(Reference) |  |
|  |  |  |  |  |  |  |  |  |  |  | 25-30 | 0.88(0.71-1.09) |  |
|  |  |  |  |  |  |  |  |  |  |  | ≥30 | 0.94(0.67-1.31) |  |
| 17 | Morimoto, 2002 | ir | US  (America) | 2.9 | 1030 | 85917 | 1993-1998 | 50-79 | 7 | Post- AND HRT Never use | ≤22.6 | 1.00(Reference) | age, education,age at menopause,parity,age at first birth,first-degree family history of breast cancer,smoking,age at menarche,race,alcohol,physical activity. |
|  |  |  |  |  |  |  |  |  |  |  | 22.6-24.9 | 1.52(0.95-2.42) |  |
|  |  |  |  |  |  |  |  |  |  |  | 24.9-27.4 | 1.40(0.87-2.23) |  |
|  |  |  |  |  |  |  |  |  |  |  | 27.4-31.1 | 1.70(1.08-2.68) |  |
|  |  |  |  |  |  |  |  |  |  |  | ＞31.1 | 2.52(1.62-3.93) |  |
|  |  |  |  |  |  |  |  |  |  | Post- AND HRT Ever use | ≤22.6 | 1.00(Reference) |  |
|  |  |  |  |  |  |  |  |  |  |  | 22.6-24.9 | 0.89(0.70-1.13) |  |
|  |  |  |  |  |  |  |  |  |  |  | 24.9-27.4 | 0.86(0.68-1.11) |  |
|  |  |  |  |  |  |  |  |  |  |  | 27.4-31.1 | 0.92(0.72-1.19) |  |
|  |  |  |  |  |  |  |  |  |  |  | ＞31.1 | 0.96(0.73-1.27) |  |
|  |  |  |  |  |  |  |  |  |  | Post- Among HRT never use age 50-59 | ≤22.6 | 1.00(Reference) |  |
|  |  |  |  |  |  |  |  |  |  |  | 22.6-24.9 | 3.16(1.13-8.84) |  |
|  |  |  |  |  |  |  |  |  |  |  | 24.9-27.4 | 1.83(0.60-5.63) |  |
|  |  |  |  |  |  |  |  |  |  |  | 27.4-31.1 | 2.86(0.98-8.35) |  |
|  |  |  |  |  |  |  |  |  |  |  | ＞31.1 | 4.46(1.60-12.44) |  |
|  |  |  |  |  |  |  |  |  |  | Post- Among HRT never use age 60-69 | ≤22.6 | 1.00(Reference) |  |
|  |  |  |  |  |  |  |  |  |  |  | 22.6-24.9 | 2.13(0.87-5.23) |  |
|  |  |  |  |  |  |  |  |  |  |  | 24.9-27.4 | 2.87(1.22-6.70) |  |
|  |  |  |  |  |  |  |  |  |  |  | 27.4-31.1 | 3.28(1.42-7.60) |  |
|  |  |  |  |  |  |  |  |  |  |  | ＞31.1 | 4.91(2.15-11.18) |  |
|  |  |  |  |  |  |  |  |  |  | Post- Among HRT never use age 70-79 | ≤22.6 | 1.00(Reference) |  |
|  |  |  |  |  |  |  |  |  |  |  | 22.6-24.9 | 0.93(0.46-1.85) |  |
|  |  |  |  |  |  |  |  |  |  |  | 24.9-27.4 | 0.66(0.32-1.39) |  |
|  |  |  |  |  |  |  |  |  |  |  | 27.4-31.1 | 0.87(0.42-1.78) |  |
|  |  |  |  |  |  |  |  |  |  |  | ＞31.1 | 1.1(0.53-2.29) |  |
| 18 | Opdahl,2011 | ir | Norway  (Europe) | 24.1 | 2890 | 58191 | 1961-2008 | ≥55 | 9 | Post- age 55-69 in Nulliparous women | <25 | 1.00(Reference) | age,birth cohort,county of residence,urban or rural community of residence,marital status,occupation,age at menarche. |
|  |  |  |  |  |  |  |  |  |  |  | 25-29 | 0.88(0.59-1.30) |  |
|  |  |  |  |  |  |  |  |  |  |  | ≥30 | 1.09(0.64-1.85) |  |
|  |  |  |  |  |  |  |  |  |  | Post- age 55-69 in Parous women | <25 | 1.00(Reference) |  |
|  |  |  |  |  |  |  |  |  |  |  | 25-29 | 1.13(0.94-1.36) |  |
|  |  |  |  |  |  |  |  |  |  |  | ≥30 | 1.17(0.93-1.49) |  |
|  |  |  |  |  |  |  |  |  |  | Post- age≥70 in Nulliparous women | <25 | 1.00(Reference) |  |
|  |  |  |  |  |  |  |  |  |  |  | 25-29 | 1.50(1.16-1.94) |  |
|  |  |  |  |  |  |  |  |  |  |  | ≥30 | 1.76(1.27-2.44) |  |
|  |  |  |  |  |  |  |  |  |  | Post- age≥70 in Parous women | <25 | 1.00(Reference) |  |
|  |  |  |  |  |  |  |  |  |  |  | 25-29 | 1.17(1.02-1.34) |  |
|  |  |  |  |  |  |  |  |  |  |  | ≥30 | 1.56(1.33-1.83) |  |
| 19 | Palmer,2007 | ir | African American  (America) | 10 | 1062 | 59000 | 1995-2005 | 21-69 | 7 | Pre- | <25 | 1.00(Reference) | age,age at menarche,parity,age at first birth,age at menopause,physical activity,education,family history of breast cancer. |
|  |  |  |  |  |  |  |  |  |  |  | 25-29 | 0.88(0.71-1.10) |  |
|  |  |  |  |  |  |  |  |  |  |  | 30-34 | 0.89(0.68-1.15) |  |
|  |  |  |  |  |  |  |  |  |  |  | ≥35 | 0.72(0.54-0.96) |  |
|  |  |  |  |  |  |  |  |  |  | Post- | <25 | 1.00(Reference) |  |
|  |  |  |  |  |  |  |  |  |  |  | 25-29 | 0.72(0.56-0.92) |  |
|  |  |  |  |  |  |  |  |  |  |  | 30-34 | 0.82(0.62-1.07) |  |
|  |  |  |  |  |  |  |  |  |  |  | ≥35 | 0.78(0.58-1.05) |  |
|  |  |  |  |  |  |  |  |  |  | Post- AND HRT Never use | <25 | 1.00(Reference) |  |
|  |  |  |  |  |  |  |  |  |  |  | 25-29 | 0.80(0.51-1.24) |  |
|  |  |  |  |  |  |  |  |  |  |  | 30-34 | 0.88(0.55-1.42) |  |
|  |  |  |  |  |  |  |  |  |  |  | ≥35 | 0.94(0.58-1.54) |  |
|  |  |  |  |  |  |  |  |  |  | Post- AND ER+/PR+ | <25 | 1.00(Reference) |  |
|  |  |  |  |  |  |  |  |  |  |  | 25-29 | 0.99(0.50-1.94) |  |
|  |  |  |  |  |  |  |  |  |  |  | ≥30 | 1.66(0.86-3.21) |  |
|  |  |  |  |  |  |  |  |  |  | Post- AND ER+/PR- or ER-/PR+ | <25 | 1.00(Reference) |  |
|  |  |  |  |  |  |  |  |  |  |  | 25-29 | 0.88(0.40-1.94) |  |
|  |  |  |  |  |  |  |  |  |  |  | ≥30 | 0.39(0.14-1.07) |  |
|  |  |  |  |  |  |  |  |  |  | Post- AND ER-/PR- | <25 | 1.00(Reference) |  |
|  |  |  |  |  |  |  |  |  |  |  | 25-29 | 1.02(0.47-2.18) |  |
|  |  |  |  |  |  |  |  |  |  |  | ≥30 | 0.88(0.39-1.97) |  |
| 20 | Phipps,2011 | ci | US  (America) | 7.9 | 2917 | 155723 | 1993-1998 | 50-79 | 8 | Post- AND ER+ | <23.75 | 1.00(Reference) | age,education,income,family history of BC,race,physical activity,history of mamography. |
|  |  |  |  |  |  |  |  |  |  |  | 23.75-26.89 | 1.19(1.05-1.35) |  |
|  |  |  |  |  |  |  |  |  |  |  | 26.90-31.04 | 1.17(1.03-1.33) |  |
|  |  |  |  |  |  |  |  |  |  |  | ≥31.05 | 1.39(1.22-1.58) |  |
|  |  |  |  |  |  |  |  |  |  | Post- AND triple negative | <23.75 | 1.00(Reference) |  |
|  |  |  |  |  |  |  |  |  |  |  | 23.75-26.89 | 0.99(0.67-1.46) |  |
|  |  |  |  |  |  |  |  |  |  |  | 26.90-31.04 | 1.21(0.83-1.77) |  |
|  |  |  |  |  |  |  |  |  |  |  | ≥31.05 | 1.38(0.92-1.99) |  |
|  |  |  |  |  |  |  |  |  |  | Post- ER+(among HRT never use) | <23.75 | 1.00(Reference) |  |
|  |  |  |  |  |  |  |  |  |  |  | 23.75-26.89 | 1.27(1.03-1.56) |  |
|  |  |  |  |  |  |  |  |  |  |  | 26.90-31.04 | 1.37(1.11-1.68) |  |
|  |  |  |  |  |  |  |  |  |  |  | ≥31.05 | 1.71(1.39-2.10) |  |
|  |  |  |  |  |  |  |  |  |  | Post- triple negative(among HRT never use) | <23.75 | 1.00(Reference) |  |
|  |  |  |  |  |  |  |  |  |  |  | 23.75-26.89 | 1.18(0.66-2.12) |  |
|  |  |  |  |  |  |  |  |  |  |  | 26.90-31.04 | 1.59(0.91-2.76) |  |
|  |  |  |  |  |  |  |  |  |  |  | ≥31.05 | 1.5(0.85-2.67) |  |
| 21 | Rapp,2005 | ir | Austria  (Austria) | 10.2 | 1045 | 78484 | 1985-2001 | 35-54 | 7 | All | 18.5-24.9 | 1.00(Reference) | smoking, occupation. |
|  |  |  |  |  |  |  |  |  |  |  | 25-29.9 | 0.96(0.83-1.10) |  |
|  |  |  |  |  |  |  |  |  |  |  | 30-34.9 | 1.07(0.88-1.31) |  |
|  |  |  |  |  |  |  |  |  |  |  | ≥35 | 1.01(0.72-1.42) |  |
| 22 | Reeves,2007 | ir | UK  (Europe) | 5.4 | 6808 | 1222630 | 1996-2001 | 50-64 | 8 | Pre- | <22.5 | 0.96(0.85-1.08) | age,geographical region,socioeconomic status,reproductive history,smoking,alcohol,physical activity,use of HRT. |
|  |  |  |  |  |  |  |  |  |  |  | 22.5-24.9 | 1.00(Reference) |  |
|  |  |  |  |  |  |  |  |  |  |  | 25-27.4 | 0.93(0.82-1.05) |  |
|  |  |  |  |  |  |  |  |  |  |  | 27.5-29.5 | 0.99(0.84-1.16) |  |
|  |  |  |  |  |  |  |  |  |  |  | ≥30 | 0.79(0.68-0.92) |  |
|  |  |  |  |  |  |  |  |  |  | Post- | <22.5 | 0.85(0.80-0.91) |  |
|  |  |  |  |  |  |  |  |  |  |  | 22.5-24.9 | 1.00(Reference) |  |
|  |  |  |  |  |  |  |  |  |  |  | 25-27.4 | 1.10(1.04-1.16) |  |
|  |  |  |  |  |  |  |  |  |  |  | 27.5-29.5 | 1.21(1.13-1.29) |  |
|  |  |  |  |  |  |  |  |  |  |  | ≥30 | 1.29(1.22-1.36) |  |
| 23 | Reinier,2007 | ir | American  (America) | 3.1 | 1191 (300 in situ,891 invasive) | 61844 | 1996-2001 | NA | 7 | Pre- in situ | <22.0 | 1.00(Reference) | age,family history,age at first birth,Nulliparous. |
|  |  |  |  |  |  |  |  |  |  |  | 22.0-24.9 | 0.90(0.60-1.50) |  |
|  |  |  |  |  |  |  |  |  |  |  | 25.0-27.4 | 1.10(0.60-2.00) |  |
|  |  |  |  |  |  |  |  |  |  |  | 27.5-29.9 | 1.02(0.50-2.10) |  |
|  |  |  |  |  |  |  |  |  |  |  | ≥30 | 1.01(0.50-1.90) |  |
|  |  |  |  |  |  |  |  |  |  | Pre- Invasive | <22.0 | 1.00(Reference) |  |
|  |  |  |  |  |  |  |  |  |  |  | 22.0-24.9 | 0.60(0.50-0.90) |  |
|  |  |  |  |  |  |  |  |  |  |  | 25.0-27.4 | 0.70(0.50-1.10) |  |
|  |  |  |  |  |  |  |  |  |  |  | 27.5-29.9 | 0.80(0.50-1.30) |  |
|  |  |  |  |  |  |  |  |  |  |  | ≥30 | 0.90(0.60-1.30) |  |
|  |  |  |  |  |  |  |  |  |  | Post- In situ | <22.0 | 1.00(Reference) |  |
|  |  |  |  |  |  |  |  |  |  |  | 22.0-24.9 | 1.10(0.70-1.70) |  |
|  |  |  |  |  |  |  |  |  |  |  | 25.0-27.4 | 0.90(0.50-1.50) |  |
|  |  |  |  |  |  |  |  |  |  |  | 27.5-29.9 | 1.10(0.60-1.80) |  |
|  |  |  |  |  |  |  |  |  |  |  | ≥30 | 0.80(0.50-1.40) |  |
|  |  |  |  |  |  |  |  |  |  | Post- Invasive | <22.0 | 1.00(Reference) |  |
|  |  |  |  |  |  |  |  |  |  |  | 22.0-24.9 | 1.20(0.90-1.60) |  |
|  |  |  |  |  |  |  |  |  |  |  | 25.0-27.4 | 1.40(1.00-1.80) |  |
|  |  |  |  |  |  |  |  |  |  |  | 27.5-29.9 | 1.60(1.10-2.10) |  |
|  |  |  |  |  |  |  |  |  |  |  | ≥30 | 1.90(1.40-2.50) |  |
| 24 | Song,2008 | ir | Korean  (Asia) | 8.75 | 713 | 170481 | 1994-2003 | 40-64 | 8 | Post- | <18.5 | 0.71(0.35-1.46) | age,height,smoking,alcohol,physical activity,pay level at study entry. |
|  |  |  |  |  |  |  |  |  |  |  | 18.5-20.9 | 0.83(0.59-1.17) |  |
|  |  |  |  |  |  |  |  |  |  |  | 21-22.9 | 1.00(Reference) |  |
|  |  |  |  |  |  |  |  |  |  |  | 23-24.9 | 1.29(1.02-1.65) |  |
|  |  |  |  |  |  |  |  |  |  |  | 25-26.9 | 1.35(1.05-1.73) |  |
|  |  |  |  |  |  |  |  |  |  |  | 27-29.9 | 1.60(1.23-2.09) |  |
|  |  |  |  |  |  |  |  |  |  |  | ≥30 | 1.86(1.25-2.76) |  |
| 25 | Sonnenschein,1999 | ci | NewYork  (America) | 6.6 | 259 | 8416 | 1985-1994 | 35-65 | 9 | Pre- | <21.5 | 1.00(Reference) | age,age at menatche,age at first full-term pregnancy,history of breast cancer. |
|  |  |  |  |  |  |  |  |  |  |  | 21.5-23.25 | 0.95(0.57-1.56) |  |
|  |  |  |  |  |  |  |  |  |  |  | 23.25-26.36 | 0.85(0.50-1.47) |  |
|  |  |  |  |  |  |  |  |  |  |  | >26.36 | 1.00(0.58-1.73) |  |
|  |  |  |  |  |  |  |  |  |  | Post- | <21.5 | 1.00(Reference) |  |
|  |  |  |  |  |  |  |  |  |  |  | 21.5-23.25 | 1.45(0.85-2.49) |  |
|  |  |  |  |  |  |  |  |  |  |  | 23.25-26.36 | 2.31(1.40-3.82) |  |
|  |  |  |  |  |  |  |  |  |  |  | >26.36 | 2.36(1.43-3.91) |  |
| 26 | Suzuki,2013 | ir | Japan  (Asia) | 12.3 | 234 | 36164 | 1988-2003 | 40-79 | 8 | Pre- | <18.5 | 0.82(0.25-2.68) | age,height,smoking,alcohol,physical activity,age at menarche,education,parity,marital status,use of HRT,family history of BC,study area. |
|  |  |  |  |  |  |  |  |  |  |  | 18.5-19.9 | 0.78(0.33-1.84) |  |
|  |  |  |  |  |  |  |  |  |  |  | 20-23.9 | 1.00(Reference) |  |
|  |  |  |  |  |  |  |  |  |  |  | 24-28.9 | 0.76(0.40-1.43) |  |
|  |  |  |  |  |  |  |  |  |  |  | ≥29 | 0.62(0.08-4.58) |  |
|  |  |  |  |  |  |  |  |  |  | Post- | <18.5 | 0.64(0.30-1.40) |  |
|  |  |  |  |  |  |  |  |  |  |  | 18.5-19.9 | 0.46(0.21-1.01) |  |
|  |  |  |  |  |  |  |  |  |  |  | 20-23.9 | 1.00(Reference) |  |
|  |  |  |  |  |  |  |  |  |  |  | 24-28.9 | 1.50(1.09-2.08) |  |
|  |  |  |  |  |  |  |  |  |  |  | ≥29 | 2.13(1.09-4.16) |  |
| 27 | Sweeney,2004 | ir | Iowa  (America) | 16 | 2286 | 36658 | 1986-2001 | 55-69 | 8 | Post- AND age of 55-64 | <23.5 | 1.00(Reference) | age,education,age at first livebirth,parity,age at menarche,family history of BC,height. |
|  |  |  |  |  |  |  |  |  |  |  | 23.5-26 | 0.86(0.64-1.16) |  |
|  |  |  |  |  |  |  |  |  |  |  | 26-29.5 | 1.26(0.96-1.64) |  |
|  |  |  |  |  |  |  |  |  |  |  | >29.5 | 1.34(1.03-1.75) |  |
|  |  |  |  |  |  |  |  |  |  | Post- AND age of 65-74 | <23.5 | 1.00(Reference) |  |
|  |  |  |  |  |  |  |  |  |  |  | 23.5-26 | 1.21(1.03-1.42) |  |
|  |  |  |  |  |  |  |  |  |  |  | 26-29.5 | 1.26(1.08-1.49) |  |
|  |  |  |  |  |  |  |  |  |  |  | >29.5 | 1.48(1.26-1.73) |  |
|  |  |  |  |  |  |  |  |  |  | Post- AND age of 75-84 | <23.5 | 1.00(Reference) |  |
|  |  |  |  |  |  |  |  |  |  |  | 23.5-26 | 1.19(0.92-1.53) |  |
|  |  |  |  |  |  |  |  |  |  |  | 26-29.5 | 1.45(1.14-1.85) |  |
|  |  |  |  |  |  |  |  |  |  |  | >29.5 | 1.44(1.12-1.84) |  |
| 28 | Tehard,2006 | ir | France  (Europe) | 3.6 years for the pre- and 4.7 years for the post- | 1135 | 69116 | 1995-2000 | 45-70 | 6 | Pre- | <18.5 | 1.15(0.47-2.79) | history of BC,age at menarche,age at first birth,parity,history of benign breast disease,alcohol,education,marital status,physical activity. |
|  |  |  |  |  |  |  |  |  |  |  | 18.5-25 | 1.00(Reference) |  |
|  |  |  |  |  |  |  |  |  |  |  | 25-30 | 0.84(0.56-1.27) |  |
|  |  |  |  |  |  |  |  |  |  |  | ≥30 | 0.26(0.06-1.00) |  |
|  |  |  |  |  |  |  |  |  |  | Post- | <18.5 | 0.49(0.22-1.10) |  |
|  |  |  |  |  |  |  |  |  |  |  | 18.5-25 | 1.00(Reference) |  |
|  |  |  |  |  |  |  |  |  |  |  | 25-30 | 1.07(0.89-1.30) |  |
|  |  |  |  |  |  |  |  |  |  |  | ≥30 | 1.44(1.04-1.99) |  |
|  |  |  |  |  |  |  |  |  |  | Post- AND HRT Never use | <18.5 | 0.61(0.25-1.48) |  |
|  |  |  |  |  |  |  |  |  |  |  | 18.5-25 | 1.00(Reference) |  |
|  |  |  |  |  |  |  |  |  |  |  | 25-30 | 0.97(0.72-1.31) |  |
|  |  |  |  |  |  |  |  |  |  |  | ≥30 | 1.40(0.91-2.17) |  |
|  |  |  |  |  |  |  |  |  |  | Post- AND HRT ever use | <18.5 | 0.63(0.31-1.27) |  |
|  |  |  |  |  |  |  |  |  |  |  | 18.5-25 | 1.00(Reference) |  |
|  |  |  |  |  |  |  |  |  |  |  | 25-30 | 1.11(0.88-1.40) |  |
|  |  |  |  |  |  |  |  |  |  |  | ≥30 | 1.45(0.90-2.33) |  |
| 29 | van den Brandt,1997 | ir | Netherlands  (Europe) | 4.3 | 626 | 62573 | 1986-1990 | 55-69 | 7 | Post- | ≤22.9 | 1.00(Reference) | Age,age at menarche,parity,age at first birth,alcohol,height. |
|  |  |  |  |  |  |  |  |  |  |  | 23-24.9 | 0.91(0.70-1.19) |  |
|  |  |  |  |  |  |  |  |  |  |  | 25-26.9 | 1.06(0.80-1.42) |  |
|  |  |  |  |  |  |  |  |  |  |  | 27-29.9 | 1.11(0.81-1.50) |  |
|  |  |  |  |  |  |  |  |  |  |  | ≥30 | 0.98(0.66-1.45) |  |
| 30 | Wada,2014 | ir | Japan  (Asia) | 11.93 | 1783 | 183940 | 1984-1996 | NA | 8 | Pre- | <19 | 1.03(0.71-1.49) | age,area,smoking,alcohol,age at menarche,age at first delivery,parity. |
|  |  |  |  |  |  |  |  |  |  |  | 19-21 | 0.94(0.74-1.18) |  |
|  |  |  |  |  |  |  |  |  |  |  | 21-23 | 1.01(0.80-1.28) |  |
|  |  |  |  |  |  |  |  |  |  |  | 23-25 | 1.00(Reference) |  |
|  |  |  |  |  |  |  |  |  |  |  | 25-27 | 1.03(0.79-1.34) |  |
|  |  |  |  |  |  |  |  |  |  |  | 27-30 | 1.36(0.79-1.34) |  |
|  |  |  |  |  |  |  |  |  |  |  | ≥30 | 1.72(1.09-2.72) |  |
|  |  |  |  |  |  |  |  |  |  | Post- | <19 | 0.54(0.39-0.74) |  |
|  |  |  |  |  |  |  |  |  |  |  | 19-21 | 0.59(0.47-0.76) |  |
|  |  |  |  |  |  |  |  |  |  |  | 21-23 | 0.81(0.68-0.97) |  |
|  |  |  |  |  |  |  |  |  |  |  | 23-25 | 1.00(Reference) |  |
|  |  |  |  |  |  |  |  |  |  |  | 25-27 | 1.03(0.78-1.37) |  |
|  |  |  |  |  |  |  |  |  |  |  | 27-30 | 0.99(0.79-1.25) |  |
|  |  |  |  |  |  |  |  |  |  |  | ≥30 | 1.27(0.88-1.83) |  |
| 31 | Weiderpass, 2004 | ci | Norway and Sweden  (Europe) | 6.7 | 733 | 99717 | 1991-1999 | 34-49 | 8 | Pre- | <20 | 1.20(0.98-1.47) | age at enrolment, parity, age at first birth, oral contraceptive use, age at menarche, family history of breast cancer, total duration of breast feeding, country of residence |
|  |  |  |  |  |  |  |  |  |  |  | 20-24.9 | 1.00(Reference) |  |
|  |  |  |  |  |  |  |  |  |  |  | 25-29.9 | 0.79(0.63-0.99) |  |
|  |  |  |  |  |  |  |  |  |  |  | ≥30 | 0.62(0.40-0.97) |  |

a. Pre-: Premenopausal women, b. Post-: Postmenopausal women, c. ER+: Estrogen receptor-positive, d. ER-: Estrogen receptor-negative, e. All: All women including premenopausal and postmenopausal women, f. PR+: Progesterone receptor-positive, g. PR-: Progesterone receptor-negative, h. HRT: Hormone replacement therapy, i. RR: relative risk, j. CI: confidence interval.
