# Supplementary material for: Body mass index had different effects on premenopausal and postmenopausal breast cancer risks: a dose-response meta-analysis with 3,318,796 subjects from 31 cohort studies
Source: BMC Public Health. 2017 Dec 8;17:936. doi: 10.1186/s12889-017-4953-9 (PMC5721381; doi:10.1186/s12889-017-4953-9)

Post never HRT use

Post ever HRT use

sub

Highest versus Lowest for pre-

Highest versus Lowest for post-

Sub between post- and pre-

Highest versus Lowest for post- ER+

Highest versus Lowest for post- ER-

Subgroup between post- ER+ and post- ER-

post- age below 65

post- age above 65

Subgroup between post- age below 65 and post- age above 65

Post- by region

Pre- by region

All 31 studies

**Pre- by follow up year**

**Post-by follow up year**

**Europe**

Europe trim and fill


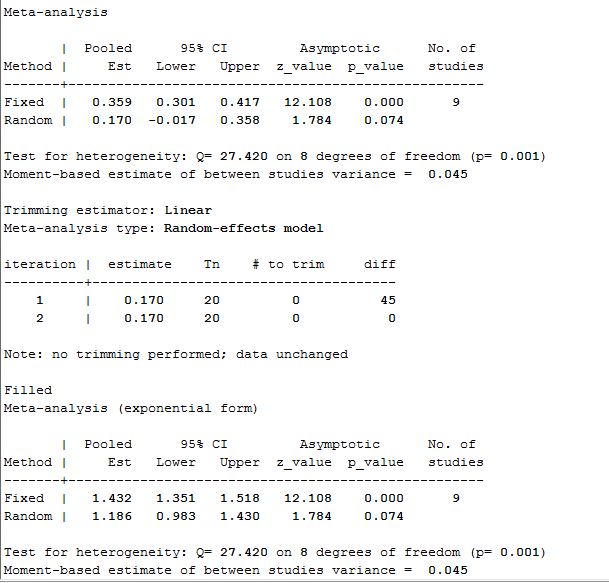


**Pre- sensitivity analysis**

metainf logrr se, random id(author) eform print


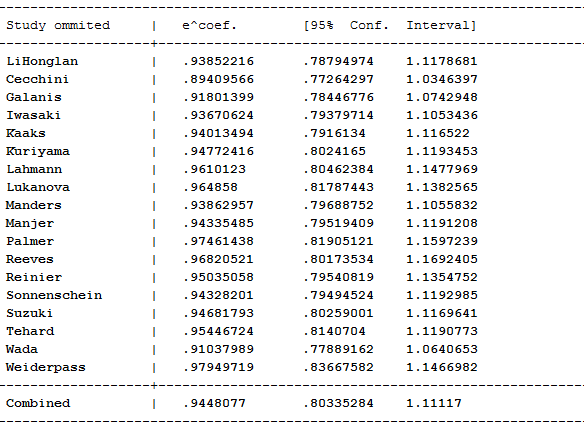


0.89(0.77-1.03)

0.98(0.84-1.15)

**Post- sensitivity analysis**

metainf logrr se, random id(author) eform print


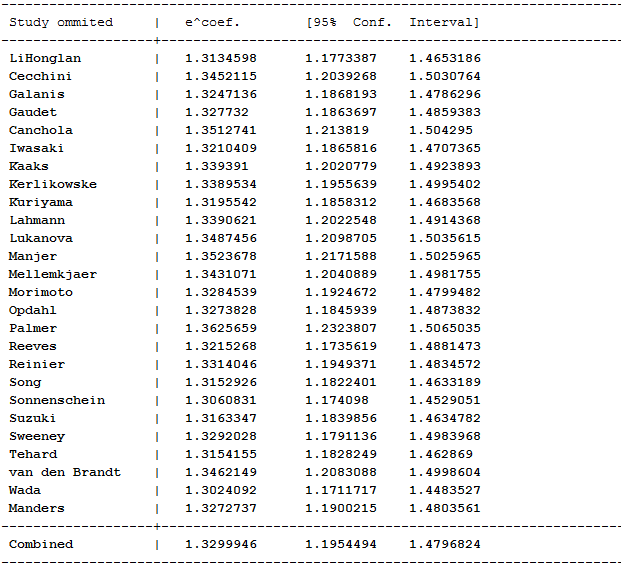


1.30(1.17-1.45)

1.36(1.23-1.51)

America

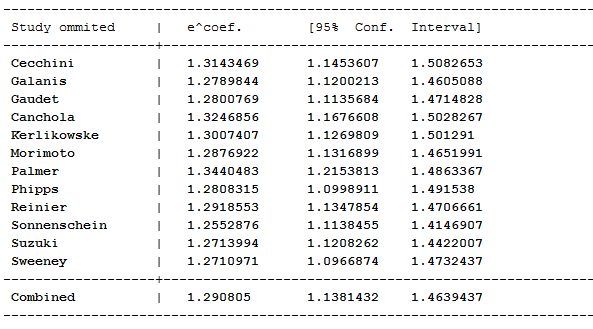


Asia

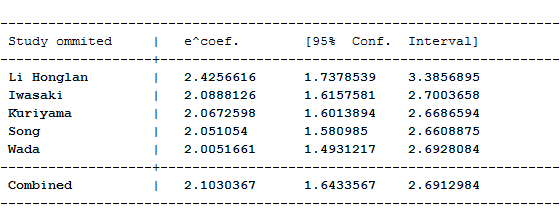


Europe

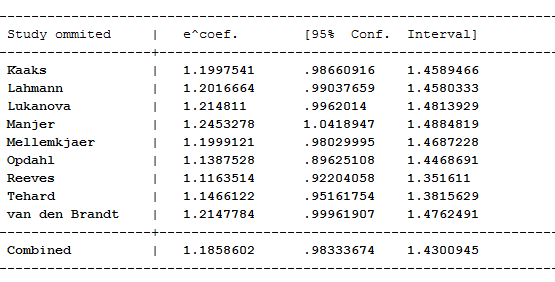

Supplement: Supplementary file 2 — The related data and materials in this study. (ZIP 1785 kb) [file 12889_2017_4953_MOESM2_ESM.zip › Forest map and sensitivity analysisR3.docx]
