# Supplementary material for: Body mass index had different effects on premenopausal and postmenopausal breast cancer risks: a dose-response meta-analysis with 3,318,796 subjects from 31 cohort studies
Source: BMC Public Health. 2017 Dec 8;17:936. doi: 10.1186/s12889-017-4953-9 (PMC5721381; doi:10.1186/s12889-017-4953-9)
Supplement: Supplementary file 2 — The related data and materials in this study. (ZIP 1785 kb) [file 12889_2017_4953_MOESM2_ESM.zip › NOS scoreR3.docx]

**Supplementary table 1 The information of Newcastle-Ottawa Scale(NOS) score**

|  |  | Selection | | | | | | | | | | | | | Comparability | | Assessment of outcome | | | | | | | | | |
| --- | --- | --- | --- | --- | --- | --- | --- | --- | --- | --- | --- | --- | --- | --- | --- | --- | --- | --- | --- | --- | --- | --- | --- | --- | --- | --- |
|  | Score | 1a | 1b | 1c | 1d | 2a | 2b | 2c | 3a | 3b | 3c | 3d | 4a | 4b | 1a | 1b | 1a | 1b | 1c | 1d | 2a | 2b | 3a | 3b | 3c | 3d |
| Li Honglan, 2006 | 8 | * |  |  |  | * |  |  | * |  |  | yes | * |  | * | * |  | * |  |  | * |  |  | * |  |  |
| Cecchini,2012 | 7 |  |  | yes^e^ |  | * |  |  |  | * |  |  | * |  | * | * | * |  |  |  | * |  |  |  |  | yes |
| Galanis,1998 | 8 |  | * |  |  | * |  |  |  |  | yes |  | * |  | * | * |  | * |  |  | * |  |  | * |  |  |
| Gaudet,2014 | 8 | * |  |  |  | * |  |  |  |  | yes |  | * |  | * | * |  | * |  |  | * |  |  | * |  |  |
| Canchola,2012 | 7 |  |  | yes^f^ |  | * |  |  |  |  | yes |  | * |  | * | * |  | * |  |  | * |  |  | * |  |  |
| Iwasaki,2007 | 9 | * |  |  |  | * |  |  |  | * |  |  | * |  | * | * |  | * |  |  | * |  |  | * |  |  |
| Kaaks,1998 | 8 | * |  |  |  | * |  |  |  | * |  |  | * |  | * | * |  | * |  |  | * |  |  |  | yes |  |
| Kerlikowske,2008 | 7 |  |  | yes^g^ |  | * |  |  |  | * |  |  | * |  | * | * |  | * |  |  |  | NA |  | * |  |  |
| Kuriyama, 2005 | 8 | * |  |  |  | * |  |  |  |  | yes |  | * |  | * | * |  | * |  |  | * |  |  | * |  |  |
| Lacey,2009 | 8 |  | * |  |  | * |  |  |  | * |  |  | * |  | * | * |  | * |  |  |  | yes |  | * |  |  |
| Lahmann,2004 | 8 | * |  |  |  | * |  |  |  | * |  |  | * |  | * | * |  | * |  |  |  | yes |  | * |  |  |
| Lukanova, 2006 | 7 | * |  |  |  | * |  |  |  |  | yes |  | * |  | * | * |  | * |  |  | * |  |  |  |  | yes |
| Lundqvist, 2007 | 7 |  |  | yes^h^ |  | * |  |  |  |  | yes |  | * |  | * | * |  | * |  |  | * |  |  | * |  |  |
| Manders,2011 | 6 |  |  | yes^i^ |  | * |  |  |  |  | yes |  |  | yes^k^ | * | * |  |  |  | yes | * |  |  | * |  |  |
| Manjer,2001 | 8 |  | * |  |  | * |  |  |  | * |  |  | * |  | * | * |  | * |  |  | * |  |  |  |  | yes |
| Mellemkjaer,2006 | 9 | * |  |  |  | * |  |  |  | * |  |  | * |  | * | * |  | * |  |  | * |  | * |  |  |  |
| Morimoto, 2002 | 7 |  | * |  |  | * |  |  |  | * |  |  | * |  | * | * |  |  | yes |  |  | yes |  | * |  |  |
| Opdahl,2011 | 9 | * |  |  |  | * |  |  |  | * |  |  | * |  | * | * |  | * |  |  | * |  |  | * |  |  |
| Palmer,2007 | 7 |  | * |  |  | * |  |  |  |  | yes |  | * |  | * | * |  |  | yes |  | * |  |  | * |  |  |
| Phipps,2011 | 8 |  | * |  |  | * |  |  |  |  | yes |  | * |  | * | * | * |  |  |  | * |  |  | * |  |  |
| Rapp,2005 | 7 |  | * |  |  | * |  |  | * |  |  |  | * |  | no | * |  | * |  |  | * |  |  |  |  | yes |
| Reeves,2007 | 8 |  | * |  |  | * |  |  |  |  | yes |  | * |  | * | * |  | * |  |  | * |  | * |  |  |  |
| Reinier,2007 | 7 |  | * |  |  | * |  |  |  |  | yes |  | * |  | * | * |  | * |  |  |  | yes |  | * |  |  |
| Song,2008 | 8 |  | * |  |  | * |  |  |  | * |  |  | * |  | * | * |  | * |  |  | * |  |  |  |  | yes |
| Sonnenschein,1999 | 9 |  | * |  |  | * |  |  |  | * |  |  | * |  | * | * |  | * |  |  | * |  |  | * |  |  |
| Suzuki,2013 | 8 | * |  |  |  | * |  |  |  |  | yes |  | * |  | * | * |  | * |  |  | * |  |  | * |  |  |
| Sweeney,2004 | 8 |  | * |  |  | * |  |  |  |  | yes |  | * |  | * | * |  | * |  |  | * |  |  | * |  |  |
| Tehard,2006 | 6 |  |  | yes^j^ |  | * |  |  |  |  | yes |  | * |  | * | * | * |  |  |  |  | yes |  | * |  |  |
| van den Brandt,1997 | 7 |  | * |  |  | * |  |  |  |  | yes |  | * |  | * | * |  | * |  |  |  | yes | * |  |  |  |
| Wada,2014 | 8 | * |  |  |  | * |  |  |  |  | yes |  | * |  | * | * | * |  |  |  | * |  |  | * |  |  |
| Weiderpass, 2004 | 8 | * |  |  |  | * |  |  |  |  | yes |  | * |  | * | * |  | * |  |  | * |  |  | * |  |  |

This scale ranged from 0 to 9 stars and awarded four stars for selection of study participants, two stars for comparability of studies, and three stars for the adequate ascertainment of outcomes.

e (women who use chemoprevention or not), f (teachers), g (women who receice mammography examinations), h(co-twin), i (BRCA1/2carrier), j(mainly teachers), k (retrospective control)
