# Supplementary material for: Body mass index had different effects on premenopausal and postmenopausal breast cancer risks: a dose-response meta-analysis with 3,318,796 subjects from 31 cohort studies
Source: BMC Public Health. 2017 Dec 8;17:936. doi: 10.1186/s12889-017-4953-9 (PMC5721381; doi:10.1186/s12889-017-4953-9)

**The code of non-linear relationship meta-analysis**

**Post-**

install.packages("rms")

library("Hmisc")

library("survival")

library("SparseM")

library("rms")

post<-read.csv("post-.csv")

knots <- quantile(post$dose, c(.1, .5, .9))

knots


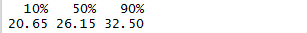


library("dosresmeta")

spl <- dosresmeta(formula = logrr ~ rcs(dose, knots), type = type, id = id,se = se, cases = cases, n = n, data = post)

summary(spl)


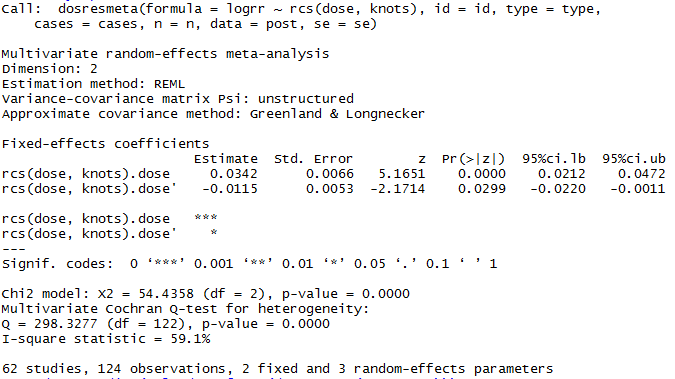


pred <- predict(spl, data.frame(dose = seq(18, 35, 1)))

pred


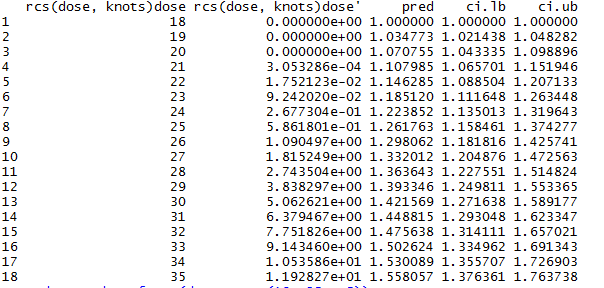


newdata = data.frame(dose = seq(18, 35, .5))

> with(predict(spl, newdata, xref = 21.5),{

+ plot(get("rcs(dose, knots)dose"), pred, type = "l", log = "y", ylab = "Relative risk", las = 1,

+ xlab = "Body Mass Index, BMI", ylim = c(.7, 2.5), bty = "l")

+ lines(get("rcs(dose, knots)dose"), ci.lb, lty = "dashed")

+ lines(get("rcs(dose, knots)dose"), ci.ub, lty = "dashed")

+ })

> rug(post$dose, quiet = T)


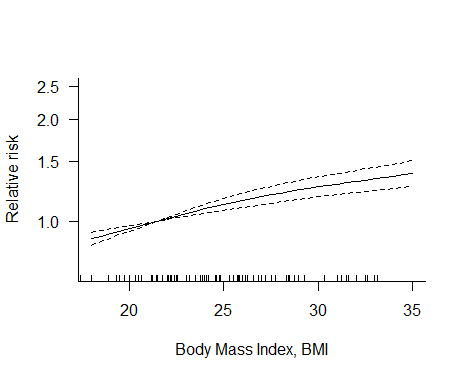


pred <- predict(spl, data.frame(dose = seq(18, 35, .5)),xref = 21.5)

pred


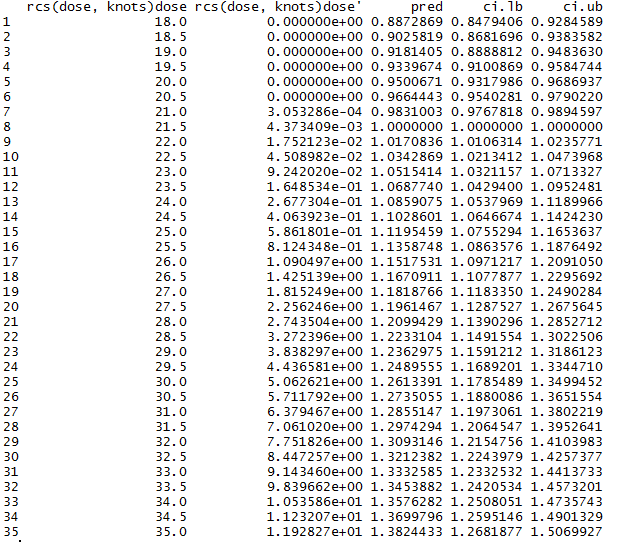


| 21.5 | 1 |
| --- | --- |
| 22.0 | 1.02(1.01-1.02) |
| 22.5 | 1.03(1.02-1.05) |
| 23.0 | 1.05(1.03-1.07) |
| 23.5 | 1.07(1.04-1.10) |
| 24.0 | 1.09(1.05-1.12) |
| 24.5 | 1.10(1.06-1.14) |
| 25.0 | 1.12(1.08-1.17) |
| 25.5 | 1.14(1.09-1.19) |
| 26.0 | 1.15(1.10-1.21) |
| 26.5 | 1.17(1.11-1.23) |
| 27.0 | 1.18(1.12-1.25) |
| 27.5 | 1.20(1.13-1.27) |
| 28.0 | 1.21(1.14-1.29) |
| 28.5 | 1.22(1.15-1.30) |
| 29.0 | 1.24(1.16-1.32) |
| 29.5 | 1.25(1.17-1.33) |

| 30.0 | 1.26(1.18-1.35) |
| --- | --- |
| 30.5 | 1.27(1.19-1.37) |
| 31.0 | 1.29(1.20-1.38) |
| 31.5 | 1.30(1.21-1.40) |
| 32.0 | 1.31(1.22-1.41) |
| 32.5 | 1.32(1.22-1.43) |
| 33.0 | 1.36(1.25-1.47) |
| 33.5 | 1.35(1.24-1.46) |
| 34.0 | 1.36(1.26-1.47) |
| 34.5 | 1.37(1.26-1.49) |
| 35.0 | 1.38(1.27-1.51) |

**Post- America**


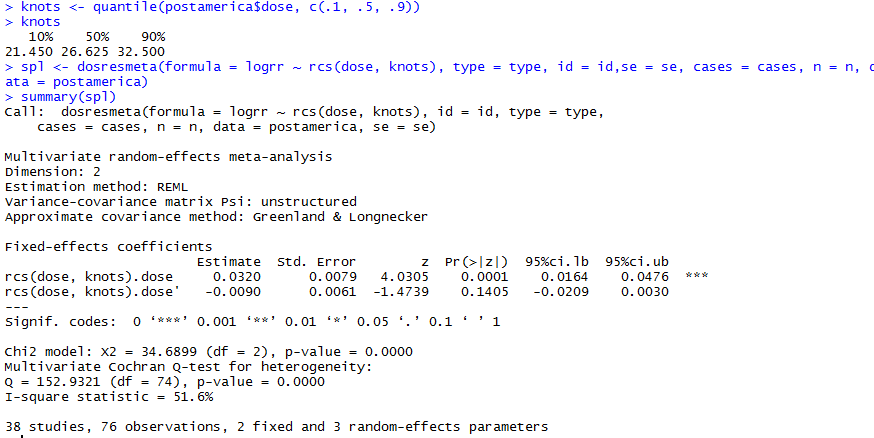


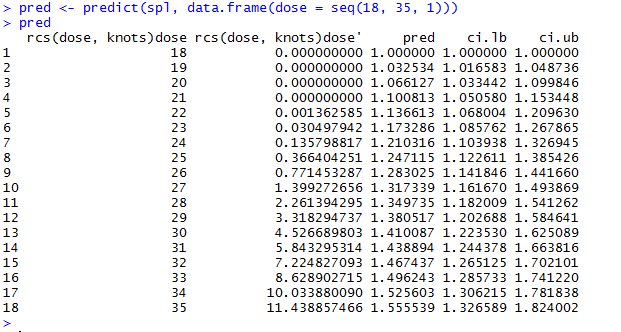


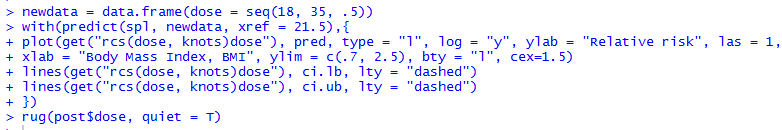


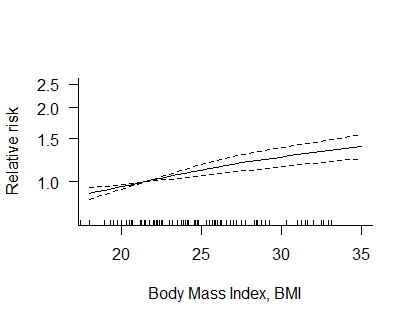


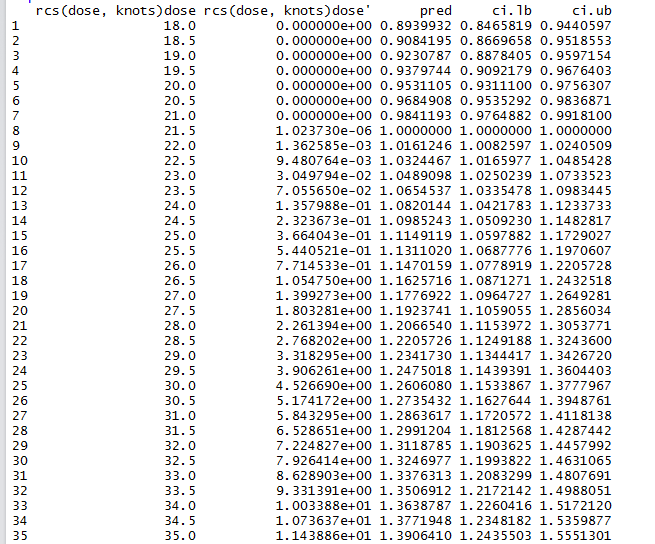


| 21.5 | 1 |  |
| --- | --- | --- |
| 22.5 | 1.03(1.02-1.05) |  |
| 23.5 | 1.07(1.03-1.10) |  |
| 25.0 | 1.11(1.06-1.17) |  |
| 26.5 | 1.16(1.09-1.24) |  |
| 30.0 | 1.26(1.15-1.38) |  |

**Post- Asia**


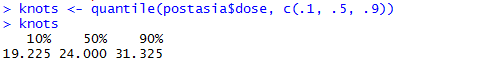


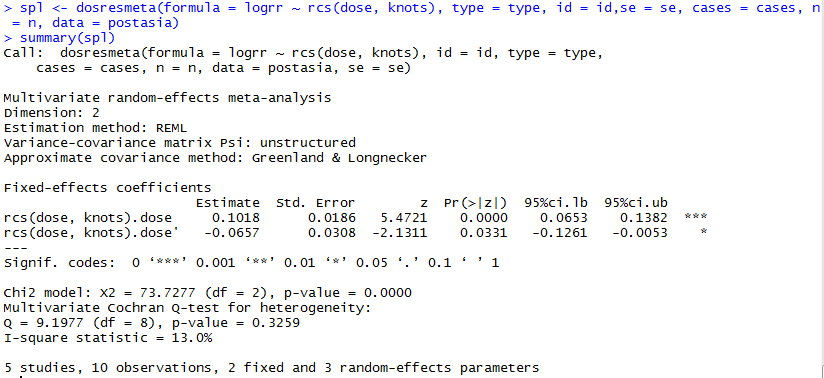


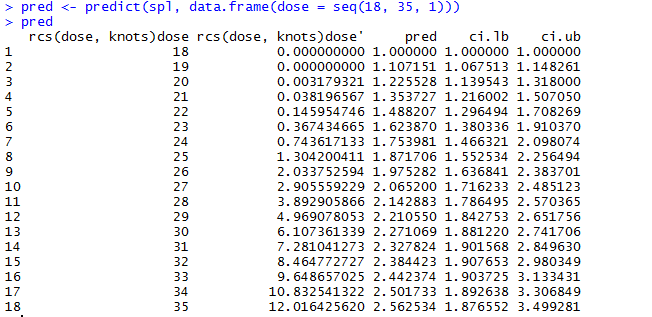


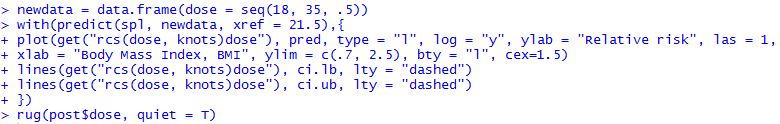


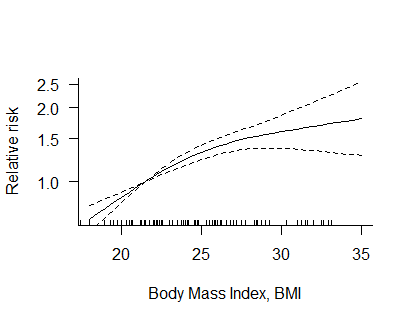


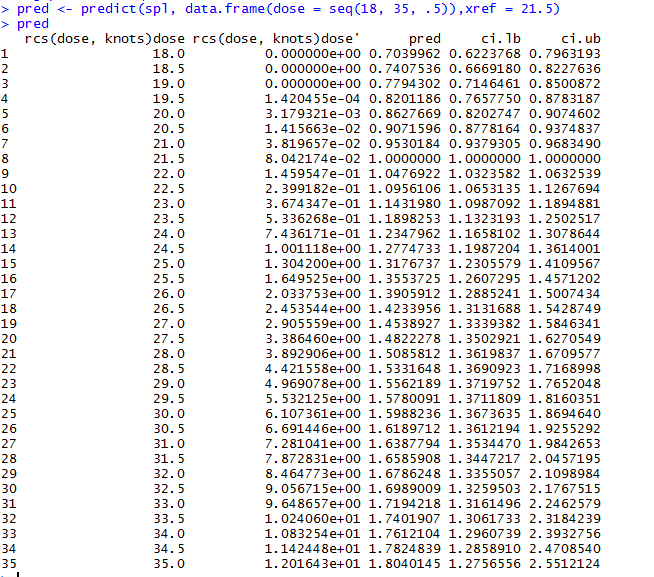


| 21.5 | 1 |
| --- | --- |
| 22.5 | 1.10(1.07-1.13) |
| 23.5 | 1.19(1.13-1.25) |
| 25.0 | 1.32(1.24-1.41) |
| 26.5 | 1.42(1.31-1.54) |
| 30.0 | 1.60(1.37-1.87) |

**Post- Europe**


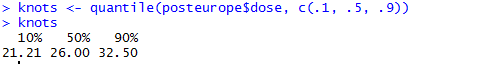


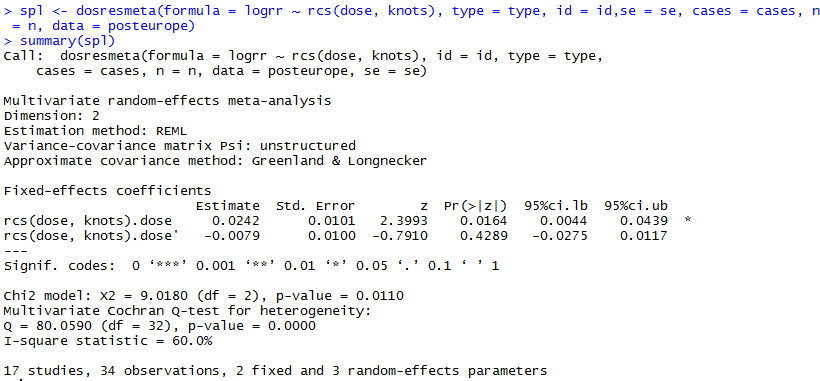


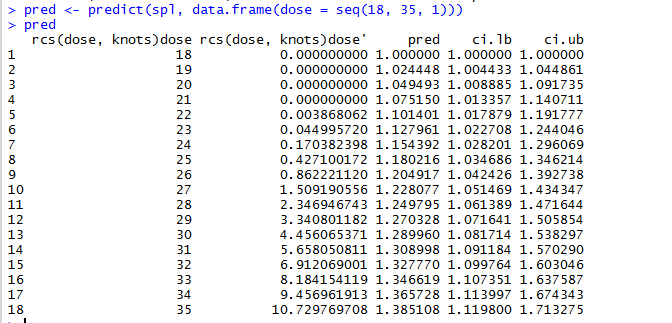


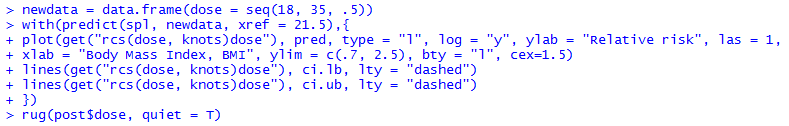


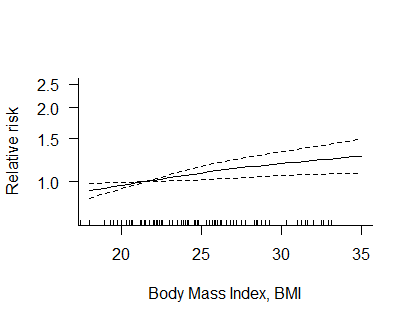


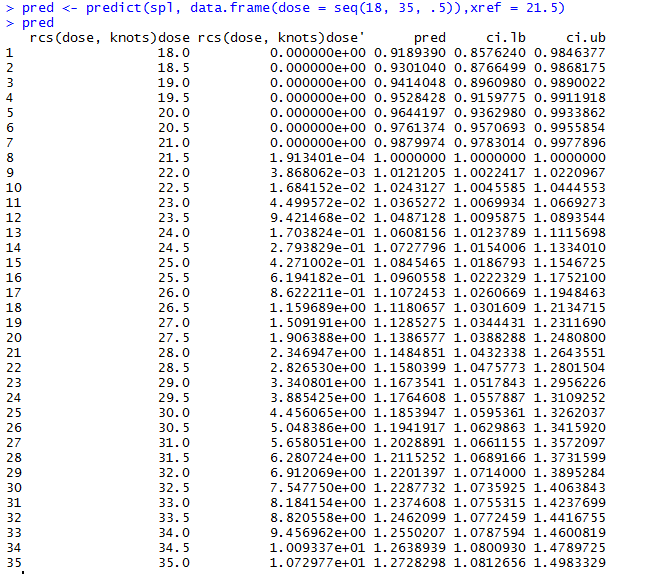


| 21.5 | 1 |
| --- | --- |
| 22.5 | 1.02(1.00-1.04) |
| 23.5 | 1.04(1.01-1.09) |
| 25.0 | 1.08(1.02-1.15) |
| 26.5 | 1.12(1.03-1.21) |
| 30.0 | 1.19(1.06-1.33) |

**Post- ER+**


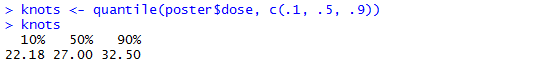


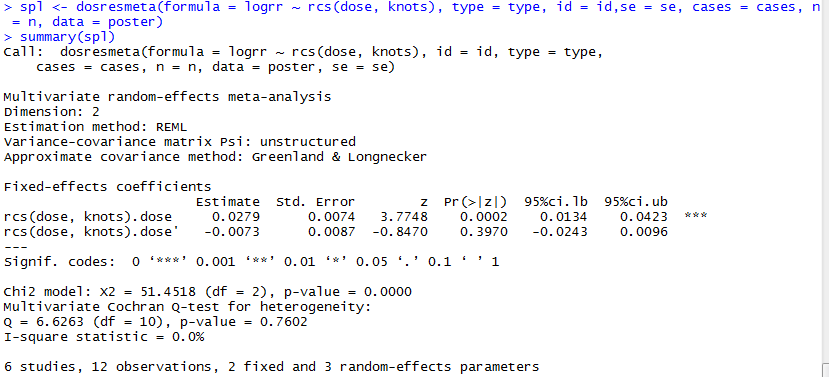


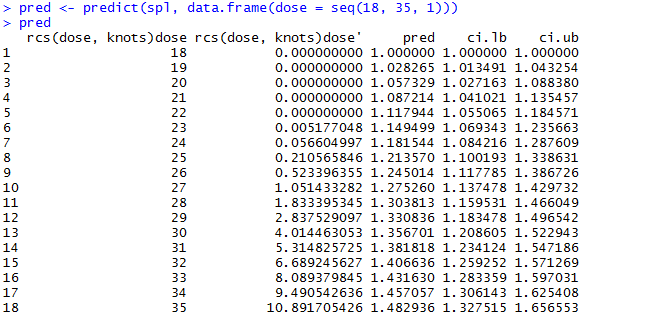


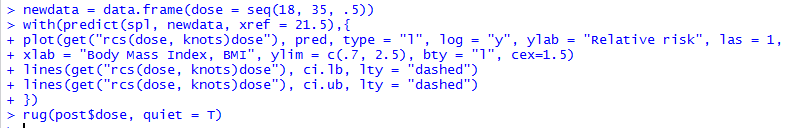


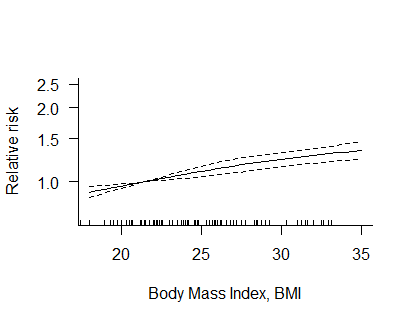


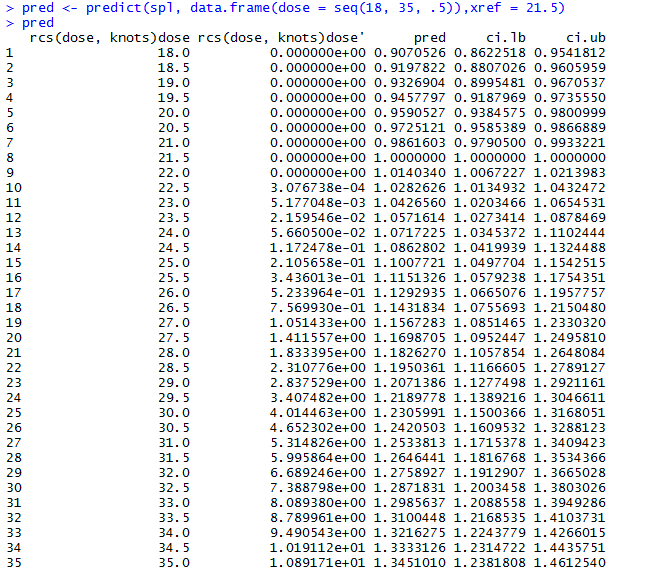


| 21.5 | 1 |
| --- | --- |
| 22.5 | 1.03(1.01-1.04) |
| 23.5 | 1.06(1.03-1.09) |
| 25.0 | 1.10(1.05-1.15) |
| 26.5 | 1.14(1.08-1.22) |
| 30.0 | 1.23(1.15-1.32) |

**Pre-**


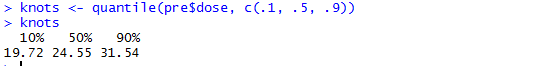


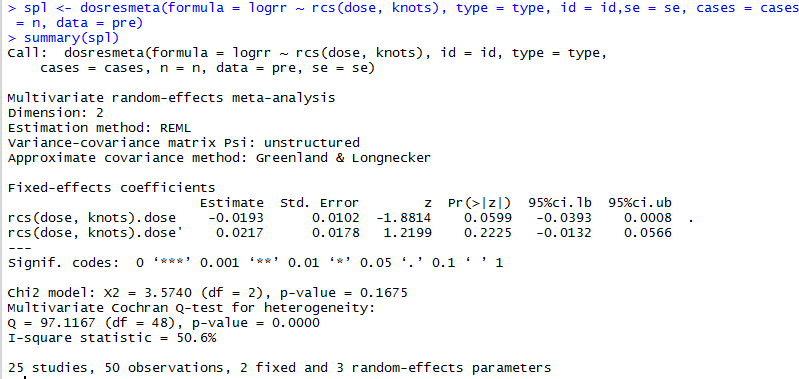


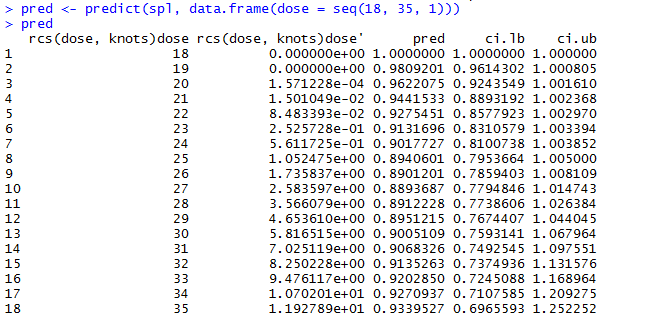


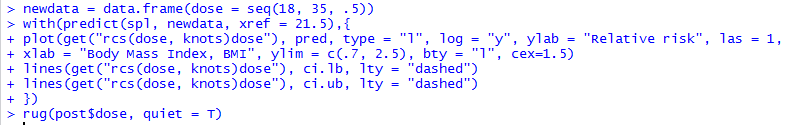


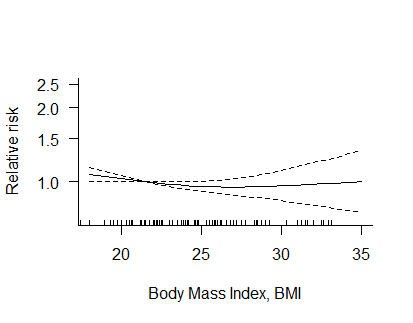


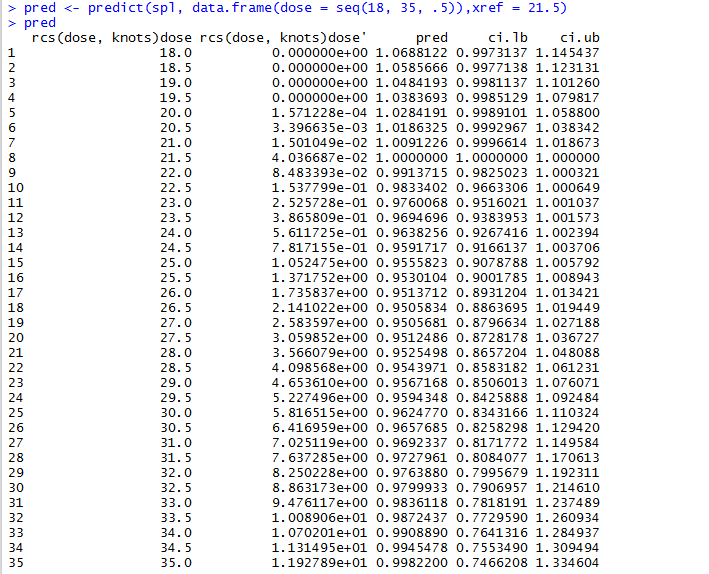


**Pre-America**


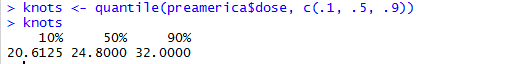


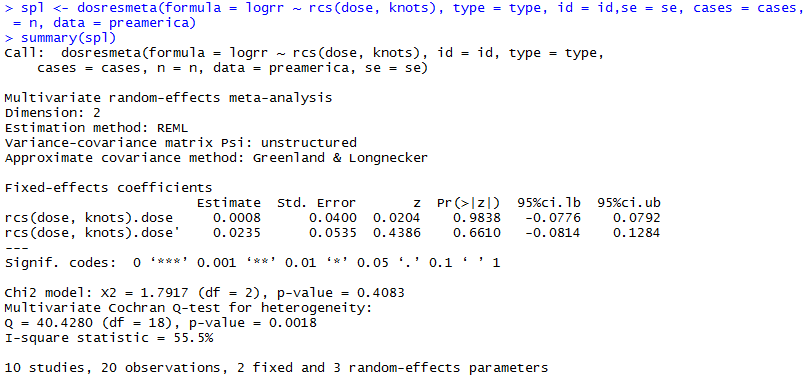


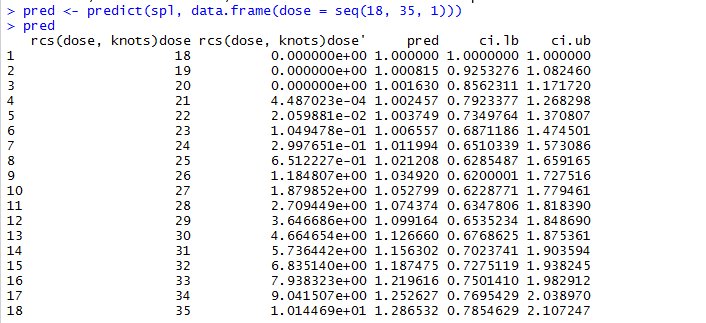


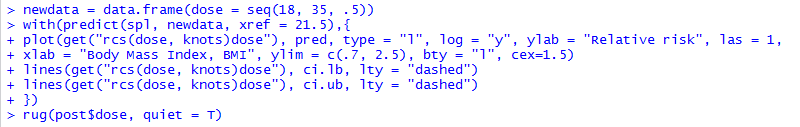


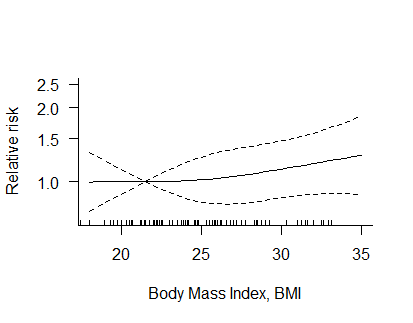


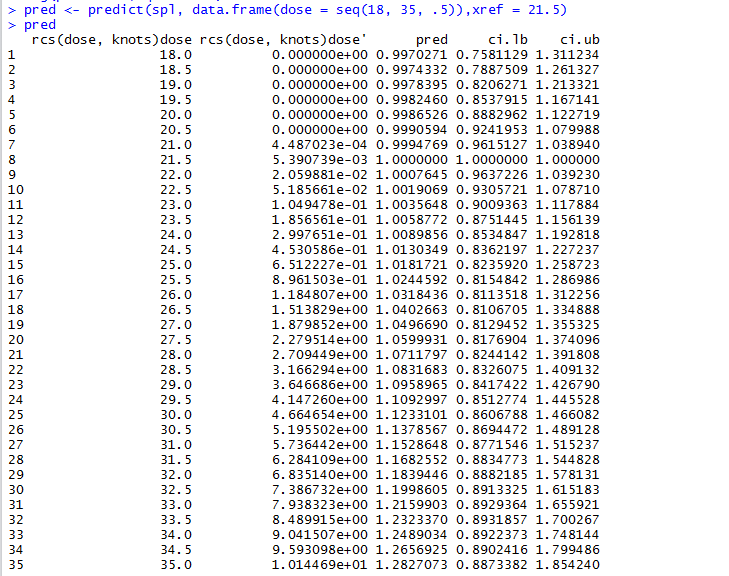


Pre- Asia


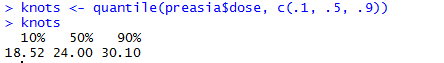


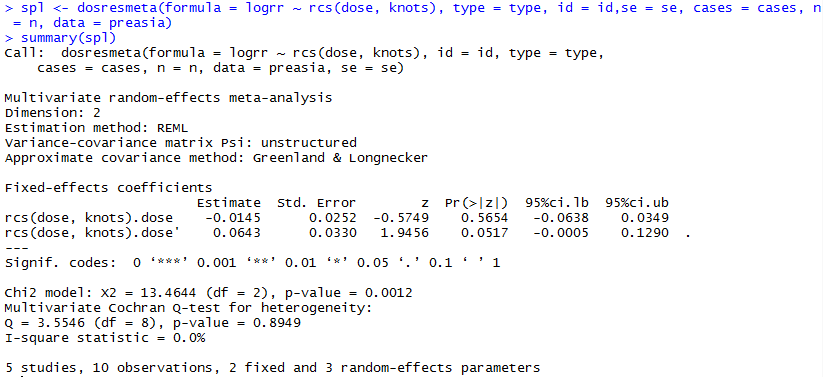


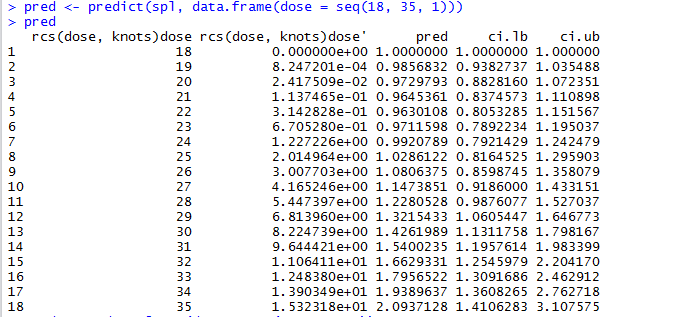


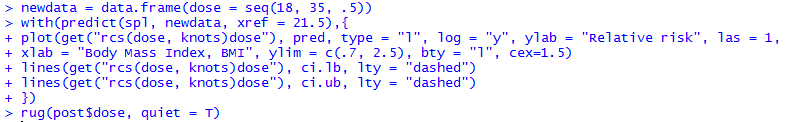


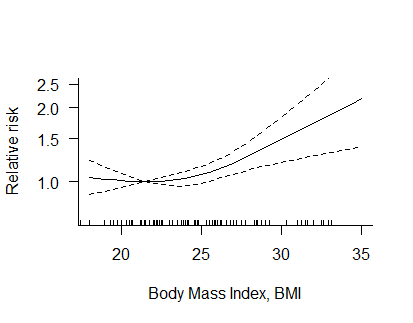


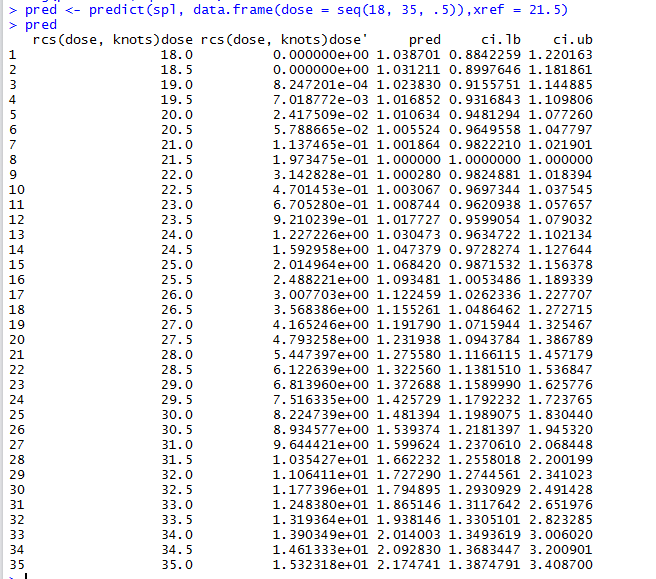


| 21.5 | 1 |
| --- | --- |
| 22.5 | 1.00(0.97-1.04) |
| 23.5 | 1.02(0.96-1.08) |
| 25.0 | 1.07(0.99-1.16) |
| 26.5 | 1.16(1.05-1.27) |
| 30.0 | 1.48(1.20-1.83) |

**Pre- Europe**


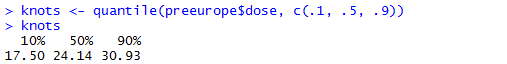


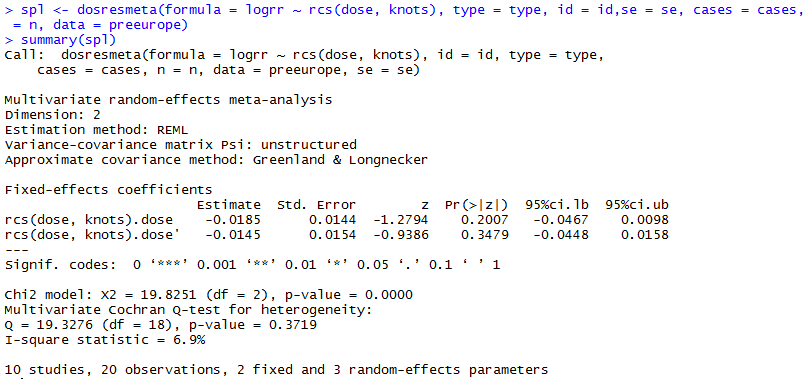


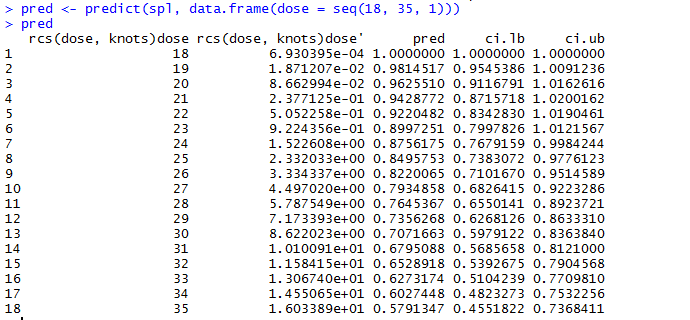


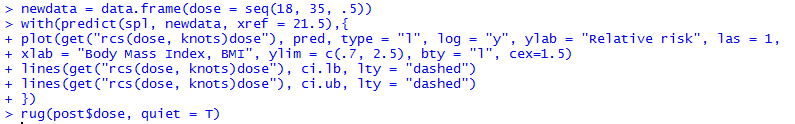


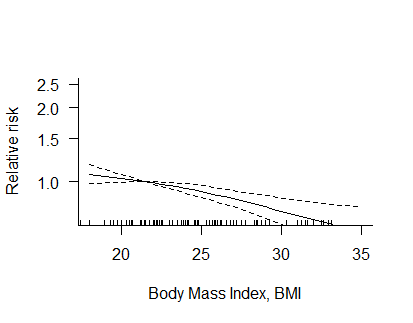


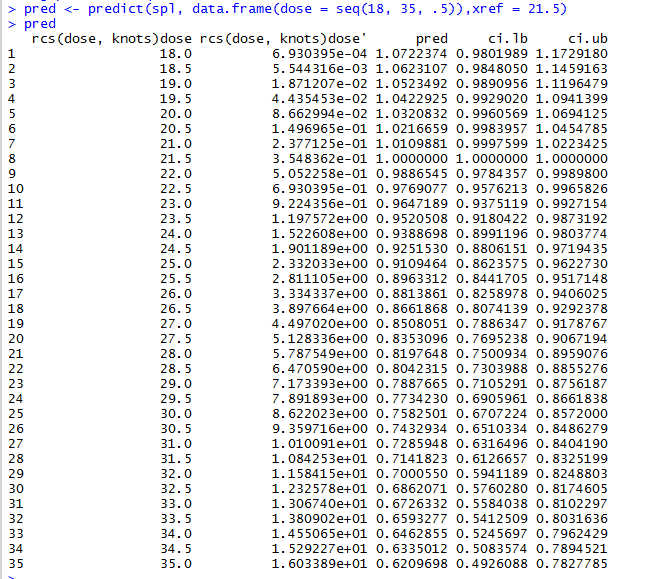


| 21.5 | 1 |
| --- | --- |
| 22.5 | 0.98(0.96-1.00) |
| 23.5 | 0.95(0.92-0.99) |
| 25.0 | 0.91(0.86-0.96) |
| 26.5 | 0.87(0.81-0.93) |
| 30.0 | 0.76(0.67-0.86) |

**Post- HRT never use**


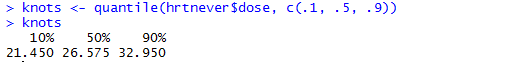


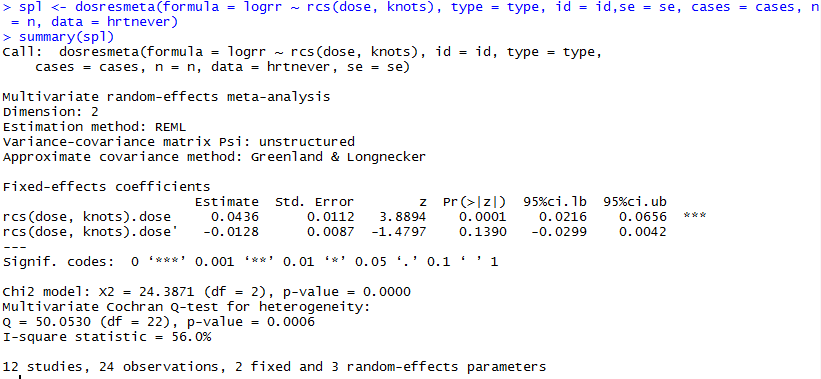


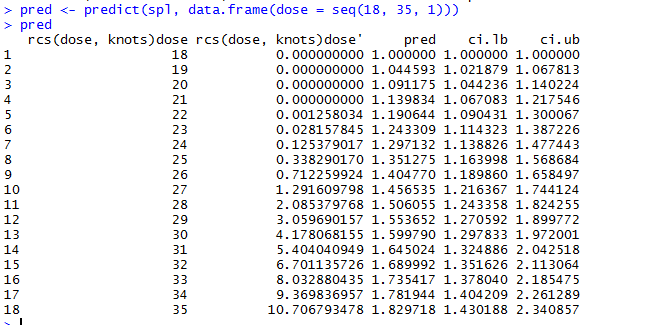


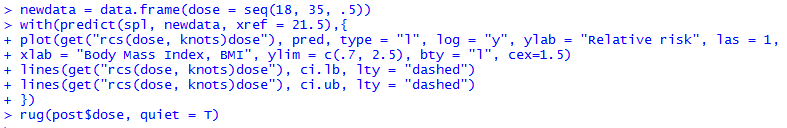


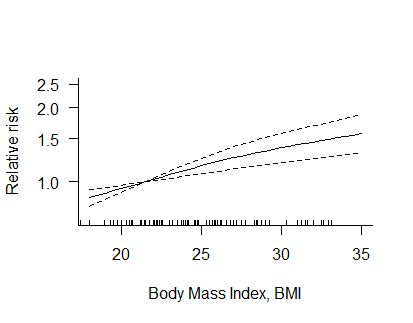


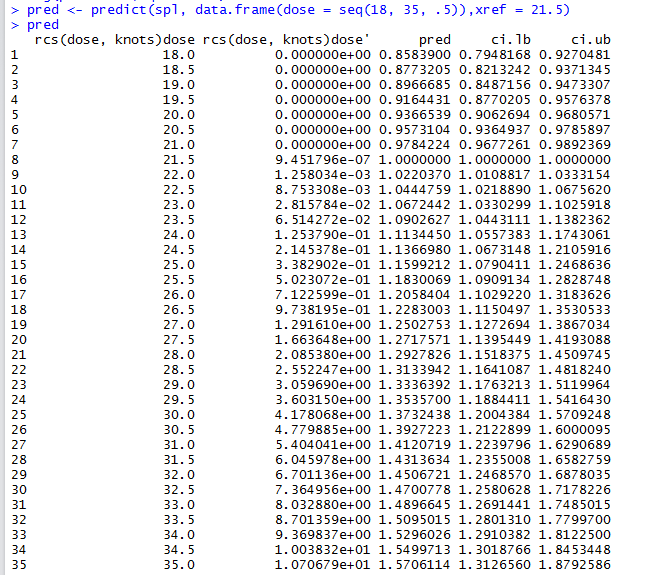


| 21.5 | 1 |
| --- | --- |
| 22.5 | 1.04(1.02-1.07) |
| 23.5 | 1.09(1.04-1.14) |
| 25.0 | 1.16(1.08-1.25) |
| 26.5 | 1.23(1.12-1.35) |
| 30.0 | 1.37(1.20-1.57) |

**Post- HRT ever use-**


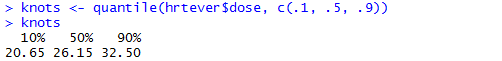


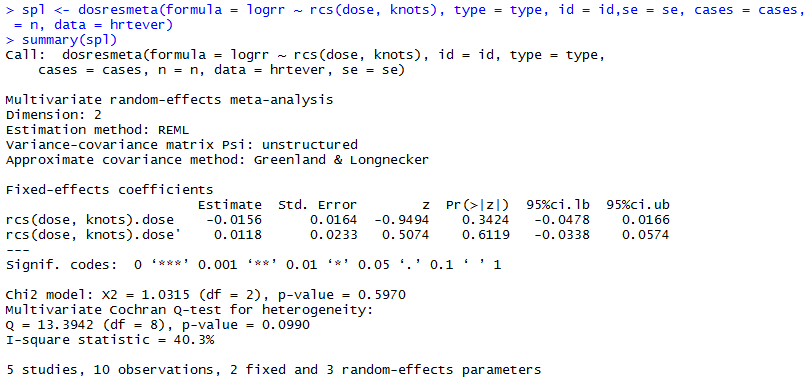


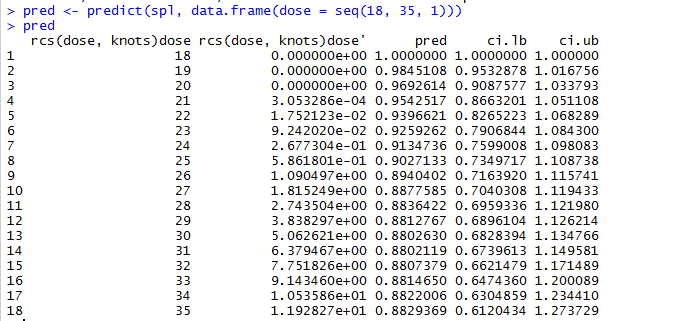


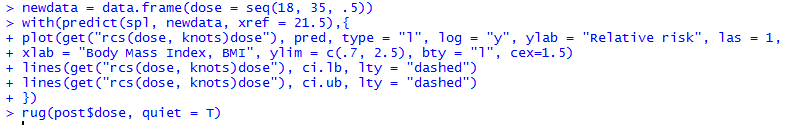


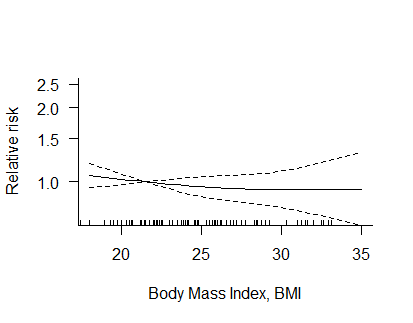


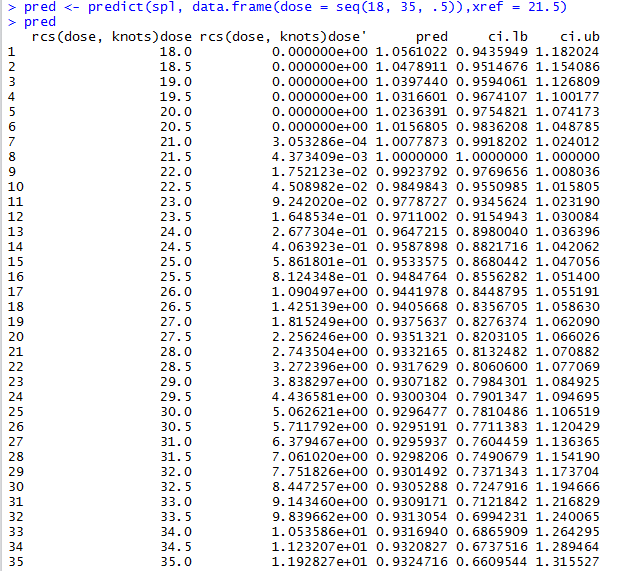

Supplement: Supplementary file 2 — The related data and materials in this study. (ZIP 1785 kb) [file 12889_2017_4953_MOESM2_ESM.zip › The code of non-linear relationship(The latest)R3.docx]
